# Hybrid 2D–CMOS microchips for memristive applications

**DOI:** 10.1038/s41586-023-05973-1

**Published:** 2023-03-27

**Authors:** Kaichen Zhu, Sebastian Pazos, Fernando Aguirre, Yaqing Shen, Yue Yuan, Wenwen Zheng, Osamah Alharbi, Marco A. Villena, Bin Fang, Xinyi Li, Alessandro Milozzi, Matteo Farronato, Miguel Muñoz-Rojo, Tao Wang, Ren Li, Hossein Fariborzi, Juan B. Roldan, Guenther Benstetter, Xixiang Zhang, Husam N. Alshareef, Tibor Grasser, Huaqiang Wu, Daniele Ielmini, Mario Lanza

**Affiliations:** 1 Materials Science and Engineering Program, Physical Science and Engineering Division, King Abdullah University of Science and Technology (KAUST), Thuwal, Saudi Arabia; 2 Institute of Microelectronics, Tsinghua University, Beijing, China; 3 Department of Electronics, Information and Bioengineering, Politecnico of Milan, Milan, Italy; 4 Department of Thermal and Fluid Engineering, Faculty of Engineering Technology, University of Twente, Enschede, the Netherlands; 5 Institute of Micro and Nanotechnology, IMN-CNM, CSIC (CEI UAM+CSIC), Madrid, Spain; 6 Institute of Functional Nano and Soft Materials, Collaborative Innovation Center of Suzhou Nanoscience and Technology, Soochow University, Suzhou, China; 7 Computer, Electrical and Mathematical Sciences and Engineering Division, King Abdullah University of Science and Technology, Thuwal, Saudi Arabia; 8 Department of Electronics and Computer Technology, Faculty of Sciences, University of Granada, Granada, Spain; 9 Department of Electrical Engineering and Media Technology, Deggendorf Institute of Technology, Deggendorf, Germany; 10 Institute for Microelectronics, TU Wien, Vienna, Austria

**Keywords:** Two-dimensional materials, Electrical and electronic engineering

## Abstract

Exploiting the excellent electronic properties of two-dimensional (2D) materials to fabricate advanced electronic circuits is a major goal for the semiconductor industry^1,2^. However, most studies in this field have been limited to the fabrication and characterization of isolated large (more than 1 µm^2^) devices on unfunctional SiO_2_–Si substrates. Some studies have integrated monolayer graphene on silicon microchips as a large-area (more than 500 µm^2^) interconnection^3^ and as a channel of large transistors (roughly 16.5 µm^2^) (refs. ^4,5^), but in all cases the integration density was low, no computation was demonstrated and manipulating monolayer 2D materials was challenging because native pinholes and cracks during transfer increase variability and reduce yield. Here, we present the fabrication of high-integration-density 2D–CMOS hybrid microchips for memristive applications—CMOS stands for complementary metal–oxide–semiconductor. We transfer a sheet of multilayer hexagonal boron nitride onto the back-end-of-line interconnections of silicon microchips containing CMOS transistors of the 180 nm node, and finalize the circuits by patterning the top electrodes and interconnections. The CMOS transistors provide outstanding control over the currents across the hexagonal boron nitride memristors, which allows us to achieve endurances of roughly 5 million cycles in memristors as small as 0.053 µm^2^. We demonstrate in-memory computation by constructing logic gates, and measure spike-timing dependent plasticity signals that are suitable for the implementation of spiking neural networks. The high performance and the relatively-high technology readiness level achieved represent a notable advance towards the integration of 2D materials in microelectronic products and memristive applications.

## Abstract (web summary)

High-integration-density 2D–CMOS hybrid microchips for memristive applications are made demonstrating in-memory computation and electrical response suitable for the implementation of spiking neural networks representing an advance towards integration of 2D materials in microelectronic products and memristive applications.

## Main

**Fig. 1:**
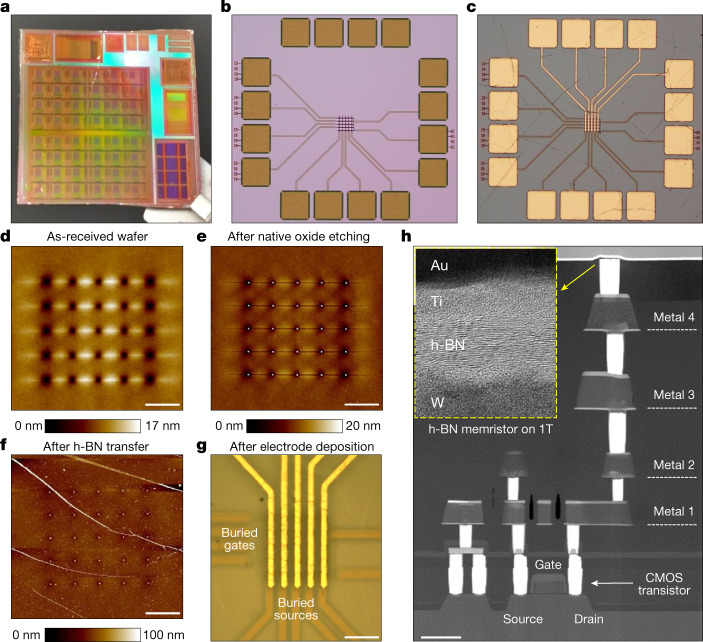
Fabrication of hybrid 2D–CMOS memristive microchips. **a**, Photograph of the 2 cm × 2 cm microchips containing the CMOS circuitry. **b**,**c**, Optical microscope images of a part of the microchip containing a 5 × 5 crossbar array of 1T1 M cells, as received (**b**) and after fabrication (**c**). The size of the squared pads is 50 μm  × 50 µm. **d**–**f**, Topographic maps collected with atomic force microscopy of the vias in the 5 × 5 crossbar arrays on the wafers as received (**d**), after native-oxide etching (**e**) and after the transfer of the h-BN sheet (**f**). **g**, Optical microscope image of a finished 5 × 5 crossbar array of 1T1M, that is, after h-BN transfer and top electrodes deposition. **h**, High-angle annular dark-field cross-sectional scanning transmission electron microscope image of a 1T1M cell in the crossbar array. The inset, which is 20 nm × 16 nm, shows a cross-sectional TEM image of the Au–Ti–h-BN–W memristor on the via; the correct layered structure of h-BN can be seen. Scale bars, **d**–**f**, 10 μm; **g**, 25 μm; **h**, 600 nm.

**Extended Data Fig. 1:**
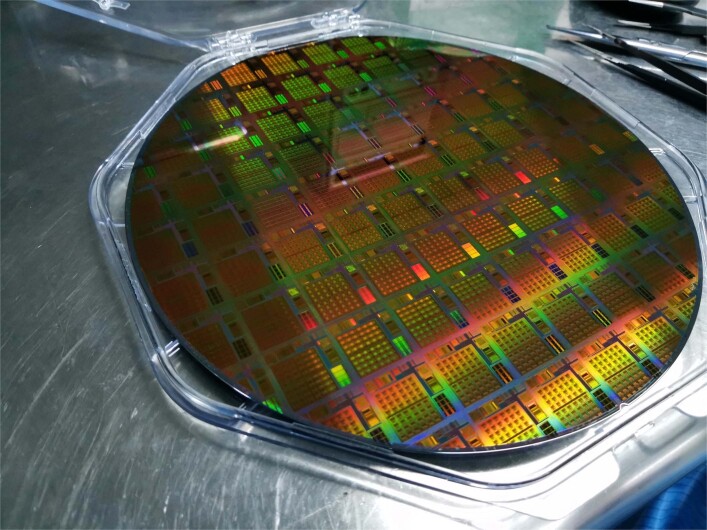
As-received silicon wafers. Photo of the 200 mm wafer received from the foundry containing all the 2 cm × 2 cm microchips, made of CMOS circuits.

Our 2 cm × 2 cm silicon microchips have been designed by means of Synopsys software and fabricated in a 200 mm silicon wafer in an industrial clean room using a 180 nm CMOS technology node (Fig. 1a and Extended Data Fig. 1). The circuits fabricated in this study consist of 5 × 5 crossbar arrays of one-transistor-one-memristor cells (1T1M, Fig. 1b,c and Supplementary Fig. 1), although some standalone memristors and CMOS transistors were fabricated for reference (Supplementary Fig. 2). The microchips have been designed to integrate the memristors into the back-end-of-line (BEOL) interconnections; that is, they have been terminated at the latest metallization layer (fourth in our wafer) and have been left without passivation. Hence, silicon oxide naturally grows on the wafers when they are extracted from the industrial clean room (Fig. 1d), which can be easily etched away to expose the tungsten vias (Fig. 1e and Supplementary Fig. 3). Then, a roughly 18-layer-thick sheet of hexagonal boron nitride (h-BN) (that is, roughly 6 nm), grown on a Cu substrate by means of chemical vapour deposition (CVD), was transferred on the microchips (Fig. 1f) using a low-temperature process (Methods). Finally, the h-BN on the contact pads was etched, and top electrodes made of different materials (that is, Au–Ti, Au or Ag) were patterned and deposited on the h-BN to finalize the circuits (Fig. 1g).

**Extended Data Fig. 2:**
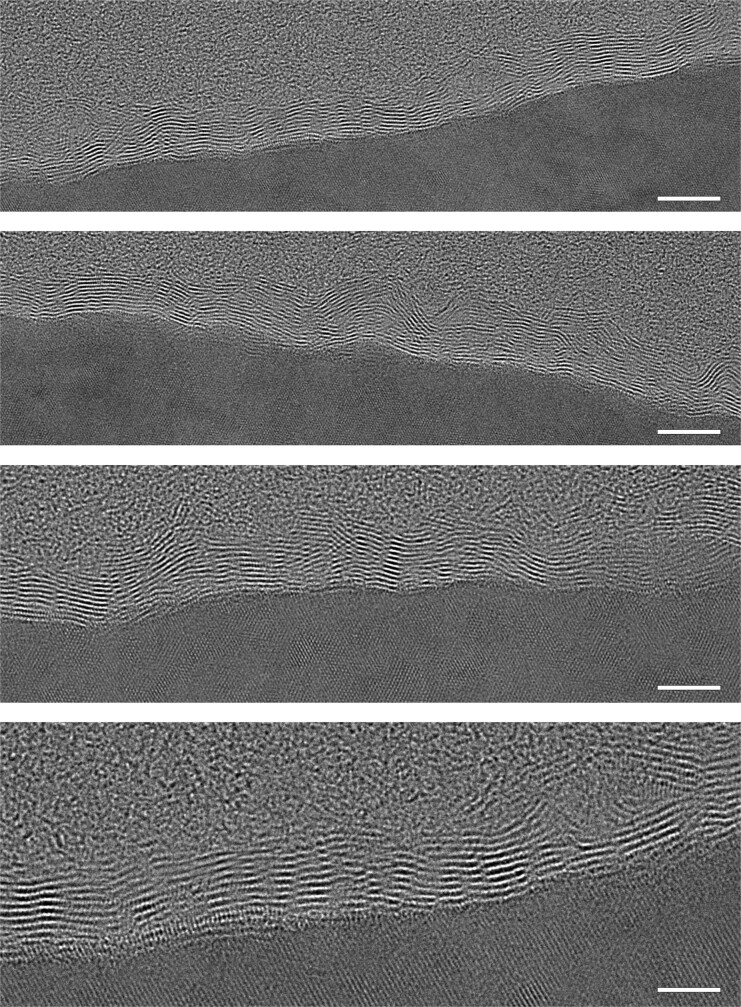
Morphological analysis of the h-BN. Cross-sectional TEM images of the as-grown multilayer h-BN sheet on Cu. The layered structure is evident, although it contains local defects (i.e., lattice distortions) produced during the CVD process – they are not related to the FIB process because our exfoliated samples never show these features. Those native defects are necessary for the observation of memristive effect. The scale bars (from top to down) and 6 nm, 6 nm, 4 nm and 3 nm.

**Extended Data Fig. 3:**
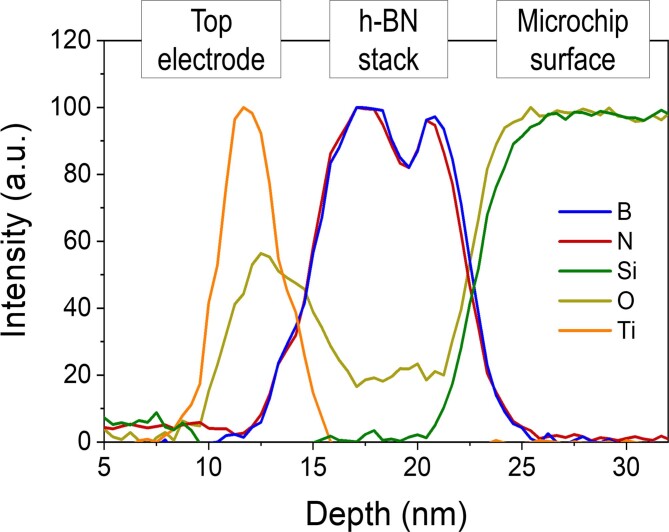
Nano-chemical characterization of the h-BN stack. Electron energy loss spectroscopy signal showing the correct structure of the h-BN stack on the CMOS microchip. The image also shows that the titanium electrode can absorb a remarkable amount of oxygen. This oxygen peak is not observed when using gold or silver electrodes.

As the tungsten vias of the fourth metallization layer have a diameter of roughly 260 nm (Fig. 1h and Supplementary Fig. 3), the lateral size of the resulting h-BN memristors is, at most, 0.053 µm^2^. Figure 1h shows a high-angle annular dark-field cross-sectional scanning transmission electron microscope image of a 1T1M cell (with top Au–Ti electrode) in the crossbar array (Supplementary Figs. 4 and 5). The correct layered structure of the h-BN stack is confirmed before and after transfer by means of cross-sectional transmission electron microscopy (TEM) (inset in Fig. 1h and Extended Data Fig. 2). Nano-chemical analyses by means of electron energy loss spectroscopy demonstrate the correct composition of the h-BN sheet (Extended Data Fig. 3). The optical microscope images (Fig. 1c) reveal that the h-BN sheet does not crack during the transfer; this is an important advantage of using roughly 6-nm-thick 2D layered materials, and it increases the yield of the devices and circuits compared to counterparts using monolayer 2D materials^6^.

## Electronic memory

**Fig. 2:**
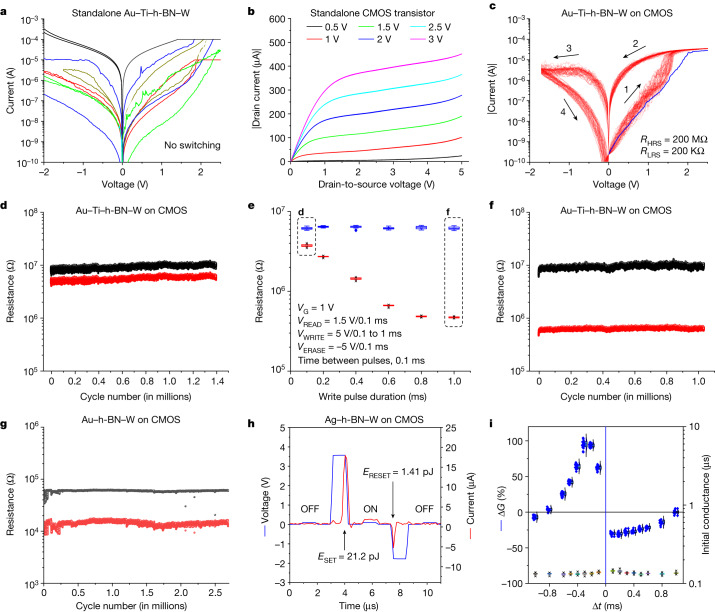
Electrical characterization of h-BN–CMOS based 1T1M cells. **a**, Electrical characterization of 0.053 µm^2^ Au–Ti–h-BN–W structures, showing erratic current fluctuations and no stable RS (each colour line corresponds to one RVS with two polarities). **b**, Typical output characteristic of all standalone CMOS transistors. **c**, Typical non-volatile bipolar RS measured in most 1T1M cells with an Au–Ti–h-BN–W memristor and a CMOS transistor (when applying *V*_G_ = 1.1 V). **d**–**f**, Endurance plots of 1T1M cell showing around 1.4 million cycles and 1 million cycles for write pulse durations of 0.1 ms (**d**) and 1 ms (**f**). **e**, *R*_LRS_ and *R*_HRS_ in a 1T1M cell when applying pulsed voltage stresses of different durations. **g**, Endurance plot showing non-volatile bipolar RS at *V*_G_ = 1 V for 1T1M cells using Au–h-BN–W memristors. All the endurance tests have been conducted following the recommended characterization process described in ref. ^42^. **h**, Voltage and current versus time in a 1T1M cell with Ag–h-BN–W memristor, showing a low switching energy. **i**, STDP characteristic of the 1T1M cell with Au–Ti–h-BN–W memristor. Before STDP characterization, the devices are always tuned to the same initial conductance (lower box charts, which relate to the right *y* axis).

When we apply sequences of ramped voltage stresses (RVS) to several standalone 0.053 µm^2^ Au–Ti–h-BN–W structures, most (roughly 90%) of them show erratic current fluctuations and no resistive switching (RS) is observed (Fig. 2a); the currents do not reach linear regime (that is, dielectric breakdown) even if we apply 11 V. This is striking because most (more than 75%) h-BN devices with larger areas (25 µm^2^) show dielectric breakdown voltages (*V*_DB_) between 3 and 11 V followed by filamentary non-volatile bipolar RS^6^. The reason should be the lower probability to find clusters of defects in small devices, which remarkably increases *V*_DB_ (ref. ^7^). Few (roughly 10%) 0.053 µm^2^ Au–Ti–h-BN–W structures show *V*_DB_ between roughly 2.5 and 4 V followed by filamentary non-volatile bipolar RS (if a current limitation 1 mA or higher is applied, Supplementary Fig. 6). However, the endurance is only around 100 cycles, mainly due to the poor controllability of the current across the memristor and the overshoot during the dielectric breakdown^6^.

**Extended Data Fig. 4:**
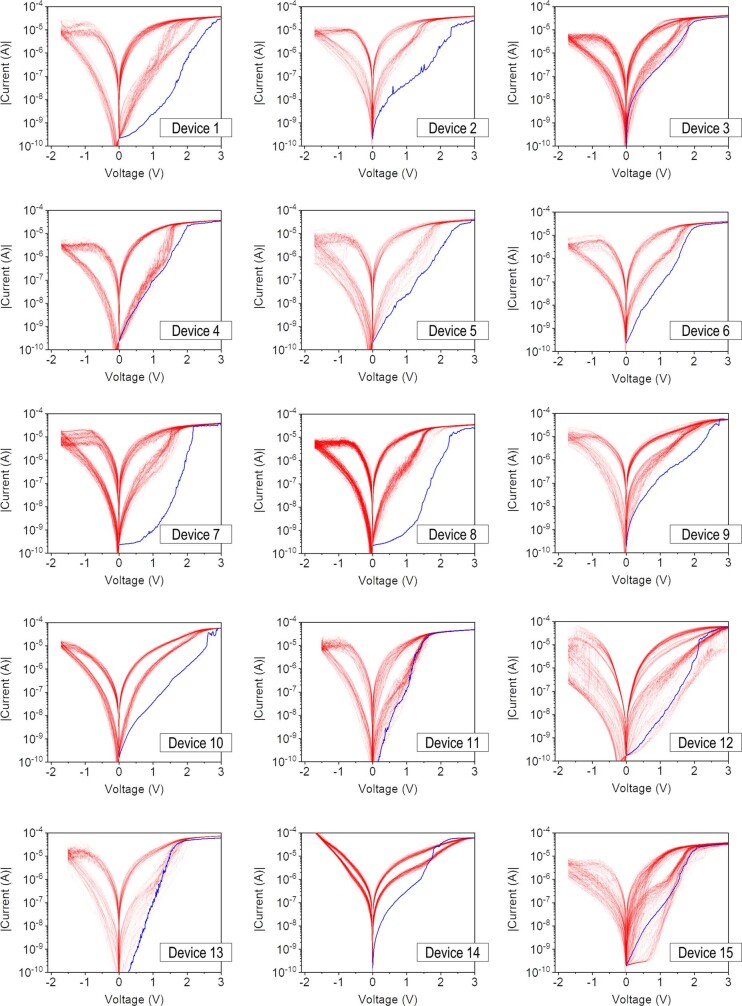
Electrical characterization of multiple h-BN memristors. Current versus voltage plots collected for different 1T1M cells using Au–Ti–h-BN–W memristors with a lateral size of 0.053 µm^2^. The plots have been drawn with 75% transparency to emphasize the general trend. Each plot contains 50 cycles or more. The first ramped voltage stress is shown in blue colour; it is normally more insulating than the rest, indicating the need of an activation step that softly degrades the h-BN and produces RS. However, no filament is completely formed (see Supplementary Note 1).

**Extended Data Fig. 5:**
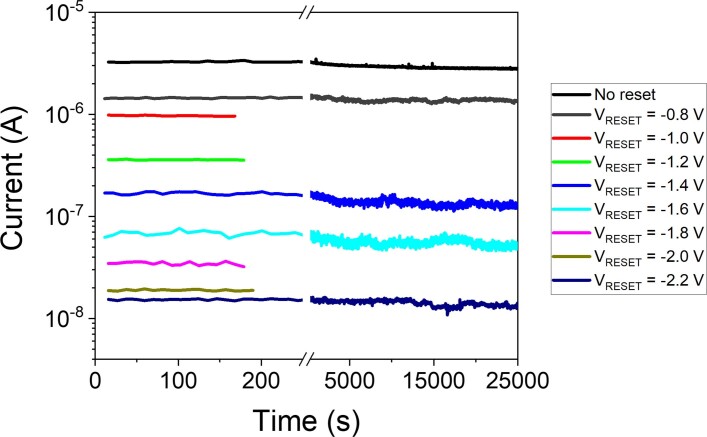
Multilevel RS in 1T1M cells. Current versus time plots for a 1T1M cell when using different end voltage of the reset RVS. Multiple stable conductance levels can be programmed. The composition of the top electrode is Au–Ti. The read voltage applied is 0.1 V. We measured the retention of several states up to 7 h, and the rest up to 200 s due to laboratory availability. The devices show no sign of conductance degradation for any measured state after 7 h, although typical drift (less than 5%) is detected, which is acceptable. According to the Semiconductor Research Corporation [V. Zhirnov, Decadal Plan for Semiconductors: New Trajectories for Memory and Storage, presented in the 2022 Non-Volatile Memory Technology Symposium 2022, December 7th-11, Stanford, USA], the unsaved bytes turnover and lifetime for multiple memory applications is currently less than 15 h, meaning that no retention time above 10 years will be needed. Similarly, the requirements in terms of retention time for memristive electronic synapses is much more relaxed than in most electronic memories, and correct functioning with short retention times of few hours has been claimed [see Kuzum, D., Yu, S., & Wong, H. P. Synaptic electronics: materials, devices and applications. Nanotechnology 24, 382001 (2013)].

On the contrary, the CMOS transistor in the 1T1M cell can precisely control the current across the h-BN memristor and avoid the current overshoot, which results in outstanding performance. First, we obtain the output characteristic of one standalone CMOS transistor by applying a constant voltage to the gate (*V*_G_) and a RVS to the drain (*V*_DS_), and measuring the drain-to-source current (*I*_DS_); the CMOS transistor works correctly as expected (Fig. 2b). And second, we measure the 1T1M cell by applying RVS at the top Au–Ti electrode of the memristor while keeping the source terminal of the transistor grounded and simultaneously applying a constant *V*_G_. When a sequence of RVS is applied to the top electrode of the Au–Ti–h-BN–W structure using *V*_G_ = 1.1 V, most 1T1M cells show non-volatile bipolar RS (Fig. 2c and Extended Data Fig. 4). The high state resistances (*R*_HRS_ of roughly 200 MΩ and *R*_LRS_ of roughly 200 KΩ)—beneficial to reduce power consumption—the non-linearity of the currents in both states and the progressive state transitions indicate that the RS is non-filamentary^8^. However, we do see an activation process, as the first RVS slightly increases the conductance of the devices (that is, softly degrades the h-BN stack, blue line in Fig. 2c and Extended Data Fig. 4). In the first microchip that we fabricated, this stable non-filamentary bipolar RS regime was observed in 32 out of 40 cells (yield 80%), and in the last one it was observed in 25 out of 25 devices (yield 100%). On the contrary, standalone Au–Ti–h-BN–W structures of 0.053 µm^2^ and 1T1M cells without h-BN never showed this behaviour; this confirms that the RS is produced by the h-BN stack and that the CMOS transistor is key to control its soft degradation. Note that in the standalone Au–Ti–h-BN–W structures the current is limited using the semiconductor parameter analyser, which activation time is long (roughly 70 µs) and parasitic capacitance is high (roughly 300 pF, related to the cables)^9^; on the contrary, in the 1T1M cell the series transistor acts as an instantaneous current limitation (it cannot drive more current than that allowed by the size of its channel) and the parasitic capacitance is much lower (roughly 50 pF, internal connections in the microchip), which reduces the duration of the switching transient and undesired currents across the Au–Ti–h-BN–W structure^10^. The values of *R*_HRS_ and *R*_LRS_ are stable over time, and multiple stable conductance levels can be programmed either by adjusting *V*_G_ during the set process (which fixes *R*_LRS_) and/or by adjusting the end voltage of the negative RVS (which fixes *R*_HRS_, Extended Data Fig. 5).

**Extended Data Fig. 6:**
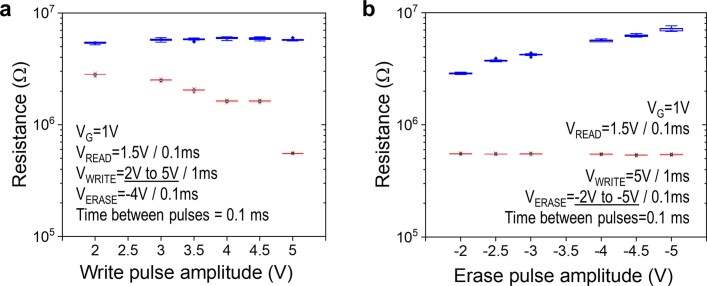
Adjustment of R_LRS_/R_HRS_ in 1T1M cells with Au–Ti top electrodes. Values of R_HRS_ and R_LRS_ depending on the pulse amplitude of write and erase operation. The value of the R_LRS_/R_HRS_ window can also be adjusted in this way.

**Extended Data Fig. 7:**
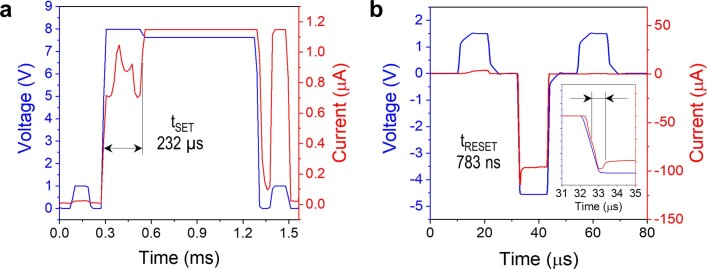
Switching time of 1T1M cells using top Au–Ti electrodes. Voltage and current versus time plots showing the non-volatile bipolar RS with high temporal resolution. The switching time for the set transition is 232 µs, and the one for the reset transition is 783 ns.

The most surprising observation, however, relates to the endurance, which readily reaches 2.5 million cycles (Fig. 2d–f) when applying sequences of pulsed voltage stresses. Under this type of stress, the values of *R*_HRS_, *R*_LRS_ and *R*_LRS_/*R*_HRS_ can be accurately controlled in three different ways: by tuning the duration of the write pulse, by tuning the amplitude of the write pulse, and by tuning the amplitude of the erase pulse (Fig. 2d–f and Extended Data Fig. 6). This endurance is very high considering the small size of the memristors (Supplementary Note 2), and similar to that of commercial metal-oxide-based resistive random access memories (0.5 million cycles)^11^ and phase-change memories (10 million cycles)^12,13^. However, the switching time of the 1T1M cells using top Au–Ti electrodes is rather long (*t*_SET_ of 232 µs and *t*_RESET_ of 783 ns, Extended Data Fig. 7).

**Extended Data Fig. 8:**
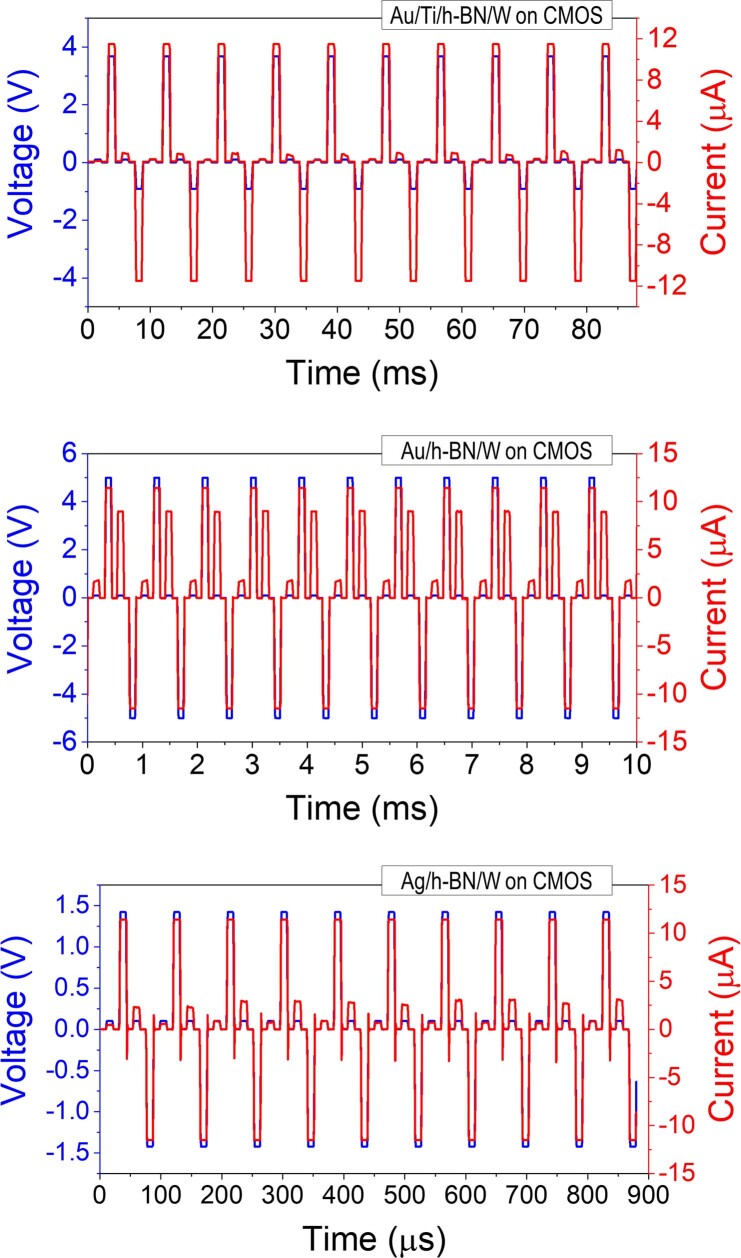
Non-volatile bipolar RS in the 1T1M cells depending on the chemical composition of the top electrode. Voltage and current versus time plots showing reproducible non-volatile bipolar RS in devices with Au–Ti, Au and Ag top electrode. The operation voltages and switching times are different, providing enough flexibility to operate in different regimes.

The properties of the 1T1M cells can be adjusted using different top electrodes (Extended Data Fig. 8). When Au electrodes are used, the devices show reliable switching at lower state resistances (Fig. 2g), as well as shorter switching time (*t*) and lower switching energy (*E*), and when Ag electrodes are used, these values can be pushed down to *t*_SET_ = 680 ns, *t*_RESET_ = 60 ns, *E*_SET_ = 21.11 pJ and *E*_RESET_ = 1.41 pJ (Fig. 2h). The reasons behind these observations are the lack of an interfacial Ti layer (which is prone to absorb oxygen, increasing the out-of-plane resistance) and the higher conductivity and diffusivity of Au^X+^ and Ag^X+^ ions^14^ (Supplementary Note 1). The performance observed in h-BN–CMOS 1T1M cells using Au–Ti electrodes may allow them to cover niche applications between NAND Flash and DRAM within the memory hierarchy (for example, persistent memory), and when using Au or Ag electrodes their performance may be valid for low-power application-specific integrated circuits within the internet-of-things^15^ (Supplementary Fig. 12).

## Data computation

**Extended Data Fig. 9:**
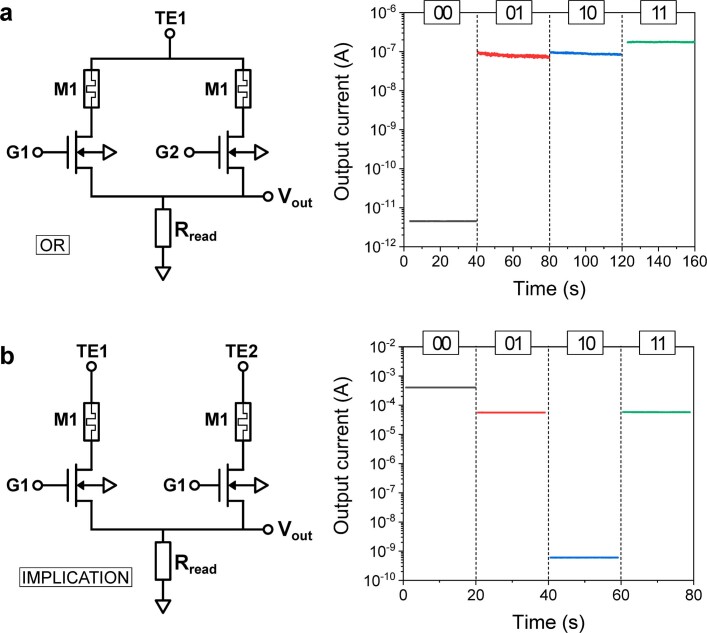
In-memory computation with crossbar arrays of h-BN memristors. **a**,**b**, Schematic and current versus time plot for “or” and “implication” logic operations (respectively) made with the crossbar array of 1T1M cells in the hybrid 2D/CMOS microchip. The value of the output current changes depending on the conductance of the memristors M1 and M2, which is indicated as “00”, “01”, “10” and “11” on each segment of the plot (“0” and “1” means low and high conductance, respectively).

On the basis of the above measured performance metrics, the hybrid 2D–CMOS 1T1M cells show good potential for data computation. The high *R*_HRS_/*R*_LRS_ ratio and the stability of the resistive states over time allows us to implement in-memory computing operations taking advantage of the internal connections of our 5 × 5 crossbar array of 2D–CMOS 1T1M cells. As a proof of concept, we realized ‘or’ and ‘implication’ operations (Extended Data Fig. 9), although more sophisticated operations could be easily realized by modifying the interconnections between the devices by means of custom design.

Furthermore, the 1T1M cells with Au–Ti–h-BN–W memristors show spike-timing dependent plasticity (STDP) when applying pairs of pulsed voltage stresses displaced in time at the input and output (Fig. 2i). This non-volatile RS performance is very attractive to construct electronic synapses for spiking neural networks (SNNs)^16^, which consume less energy than traditional deep neural networks^17^.

**Fig. 3:**
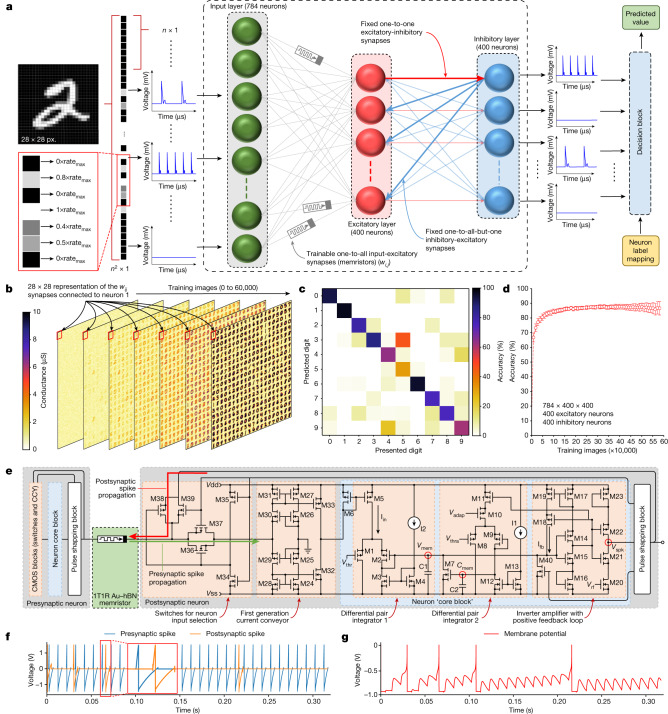
Implementation of a SNN using CMOS–h-BN based 1T1M cells. **a**, Structure of the considered SNN. Each MNIST image is reshaped as a 784 × 1 column vector, and the intensity of the pixels is encoded in terms of the firing frequency of the input neurons. The only trainable synapses are those connecting the input layer with the excitatory layer, and they are modelled with the STDP characteristic of the CMOS–h-BN based 1T1M cells. The learning is unsupervised, and the neurons are labelled only after the training. These label-neuron assignments are then feed to the decision block altogether with the firing patterns of the neurons, to infer the class of the image presented in the input. **b**, Evolution of the synaptic connections between the input and excitatory layers during training for the case of 400 excitatory and/or inhibitory neurons. The red square identifies 784 synapses arranged in a 28 × 28 representation. **c**, Confusion matrix indicating the classification accuracy for each class from the dataset. **d**, Classification accuracy as a function of the number of presented training images for the neural network comprising 400 excitatory and/or inhibitory neurons. The error bars show the standard deviation for 50 Monte Carlo simulation runs for every accuracy point. **e**, Circuit schematic of the proposed neuron–synapse–neuron block combining h-BN based 1T1M cells and CMOS circuitry. The colours indicate the complete neuron (grey surrounding box), the core block (light-blue box) and the individual building blocks (light-red boxes). CCY, current conveyor. **f**, SPICE simulation of the pre- and postsynaptic signals applied to the CMOS–h-BN based 1T1M. **g**, SPICE simulation of the neuron’s membrane potential. The firing events progressively separate from each other due to the adaptative firing threshold.

Although implementing by means of hardware a reliable 2D materials-based memristive SNN capable of competing with state-of-the-art developments^18,19^ is not yet achievable due to the lower maturity of these materials, we can analyse the performance of a SNN made of memristors that show STDP characteristics such as those in Fig. 2i (Supplementary Note 3). First, we fit the measured STDP data from Fig. 2i, including the device-to-device variability, using an exponential decaying model to implement the learning rule (Supplementary Fig. 13). Second, we simulate a SNN to demonstrate the unsupervised learning capability (Fig. 3a), and benchmark it by classifying the images from the Modified National Institute of Standards and Technology (MNIST) database of handwritten digits^20,21^ (Methods). The SNN has 784 input neurons, an excitatory layer of 400 neurons and an inhibitory layer of 400 neurons, plus a decision block that determines which is the most probable digit (0–9) represented by the input pattern. We trained the SNN with the complete MNIST dataset and evaluated the accuracy every 1,000 images. Figure 3b–d shows the main three figures-of-merit for this type of SNNs (that is, evolution of the synaptic weights with the number of training images, confusion matrix of the network and the training accuracy versus number of training images) and all of them indicate an excellent performance. To account for the device variability, we considered a Monte Carlo simulation with 50 iterations that randomizes the exponential fitting of the STDP plot and the initial value of the synapses, and the deviations observed in the accuracy are very low (less than 5%, Fig. 3d and Supplementary Fig. 14). The best average accuracy reaches roughly 90%, which is a very high value considering the simplicity of the SNN and the unsupervised training protocol (Supplementary Table 4).

We also propose a CMOS circuit for the hardware implementation of an electronic neuron based on our h-BN memristors (Fig. 3e), which is capable of accounting for the adaptative firing threshold and the refractory period after firing (pre- and postsynaptic traces and the evolution of the membrane potential, simulated by means of SPICE, in Fig. 3f,g).

## Discussion

Very few commercial electronic products today already include 2D materials, and the ones that do (sensors^22^, specialty cameras^23^) use very low integration density (more than 100 µm^2^ per device)—because in larger devices the local defects in the 2D material are not so detrimental. Our hybrid 2D–CMOS microchips are still far from being ready for production, but we can safely claim that our work represents the highest performance and technology readiness level ever achieved in high-integration-density 2D materials-based electronic devices or circuits. The electrical characteristics of the h-BN memristors connected to a CMOS transistor are by orders of magnitude superior to those of standalone h-BN memristors^6,24–27^ and h-BN memristors connected to 2D materials-based transistors^28,29^.

The voltages needed to switch our devices (from ±1.4 to ±5 V) are low compared to other prototypes in the field of 2D materials (even more than 20 V)^30–32^, but still higher than that used at the 180 nm CMOS node. Nevertheless, this is not an impediment for the development of this technology, as there are many commercial microchips that operate at much higher voltages; that is the case for all Flash memories^33^ (state-of-the-art 3D-NAND Flash memories are programmed at around 20 V)^34^ and all bipolar-CMOS microchips for automotive applications (which require up to 40 V)^35^. Strategies to fabricate wafers with devices that operate at different voltages are widespread^36^, and many companies^37,38^ offer versions of their 180 nm CMOS technology that operate at high voltages greater than 18 V. Note that prototype memristive devices developed by companies also operate at ±5 V (ref. ^39^).

**Extended Data Fig. 10:**
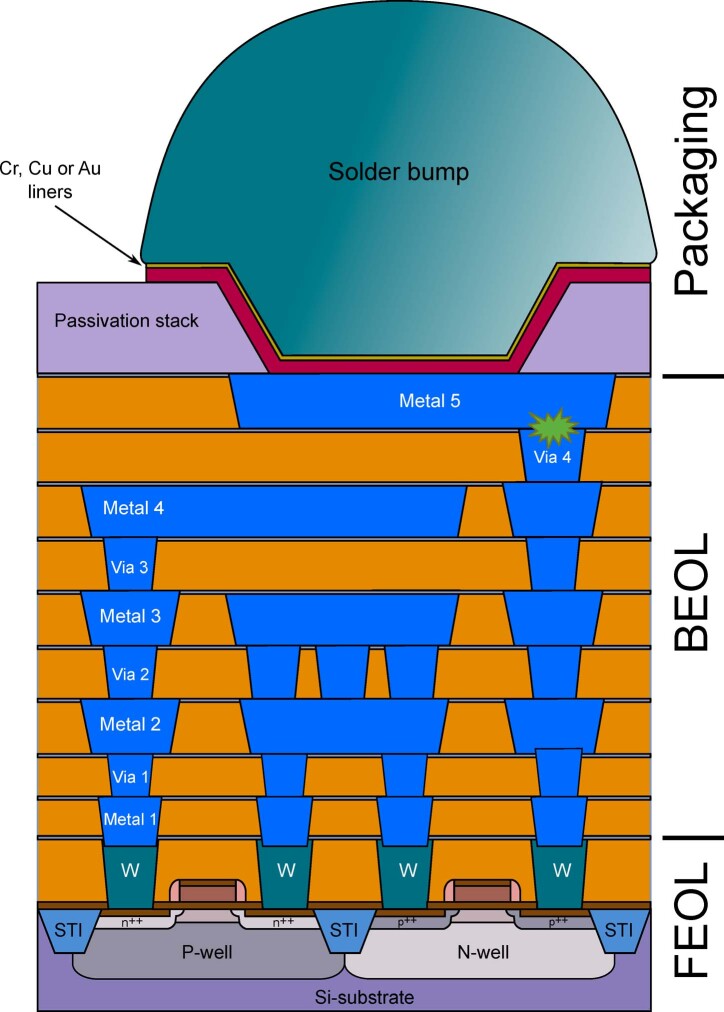
Non-exhaustive list of materials for microchip manufacturing. Schematic representation of a microchip; the position of our memristor is indicated with a green star. Our memristors are integrated in the last metallization layer of the BEOL, where Au liners are often employed, and very far from the transistors in the FEOL. Apart from this, Au wire bonding to the socket is also often employed. Hence, the use of Au electrodes of the memristors is not a concern for this type of devices/circuits. STI means shallow trench isolation.

We finally remark that, at a first glance, the use of Au and Ag electrodes may not appear ideal because they are categorized as contaminant in front-end-of-line (FEOL) processes. However, our h-BN memristors are integrated in the latest metallic layer of the BEOL interconnections (Fig. 1h), where Au pads, liners and wires are usually used^40^ (Extended Data Fig. 10). The semiconductor industry has also developed ferroelectric memories with high content of Iridium^41^ (a contaminant material forbidden in FEOL processes), and companies working in the field of 2D materials use Au electrodes in their studies and FEOL prototypes (Supplementary Table 5). Hence, the use of Au, Au–Ti or Ag electrodes in our hybrid 2D–CMOS microchips for memristive applications does not prevent their adoption by the industry.

## Methods

### Microchip fabrication

The metal-oxide-semiconductor field-effect-transistor circuits were fabricated in a standard CMOS foundry. The size of the wafers is 200 mm, and the technology node was 180 nm. Each wafer contained 60 chips with a size of 2 cm × 2 cm, and each of them contained different circuits, including the unfinished memristors, finished transistors and unfinished 5 × 5 crossbar arrays of 1T1M cells. As we received the silicon wafer without a passivation layer, first we cut it to separate the microchips and then etched the native oxide by immersing them in a diluted hydrofluoric acid solution (10:1) for 1 min to etch the native oxide (SiO_2_). This step was carried out to expose the conductive tungsten vias to make a good electrical contact with 2D materials. Second, we transferred a sheet of multilayer h-BN (previously grown by CVD on a Cu foil) using a wet transfer method. One layer of polymethyl methacrylate (PMMA) with thickness of around 300 nm was spin coated on the cut h-BN. The PMMA–h-BN–Cu sample was deposited on a FeCl_3_ solution (0.1 g ml^−1^) to etch the Cu substrate and, once the Cu disappeared, the resulting PMMA–h-BN sample was washed in diluted HCl solution (1 mol l^−1^ for 1 min) and deionized water (for 1 h). The PMMA–h-BN sample was picked up using the native oxide-free CMOS microchip and dried naturally in a dry box. The PMMA was then removed by immersing the sample in acetone for 24 h. Third, we used photolithography (mask aligner from SUSS MicroTec, model MJB4) to expose the h-BN on top of the metallic pad. Then we used a dry etching method with Ar–O_2_ plasma (Plasma Cleaner from PVA TePla America Inc., Model IoN 40) to etch the h-BN (300 W for 10 min) and expose the pads. Finally, we used photolithography, electron beam evaporation (Kurt J. Lesker, model PVD75) and a lift-off process (rinse in acetone for 1 min) to pattern and deposit the top electrodes and/or drain electrodes (3 nm Ti with 40 nm Au on top without breaking the vacuum, or 50 nm Au or 50 nm Ag). The process was simple and reproducible, although we believe it could be considerably improved if optimized methods in an industrial clean room are used. Ideally, the h-BN should be grown in large CVD systems (for example, Aixtron^43^) and transferred on the wafers before cutting them in multiple microchips, using methods like laser debonding^44^. Note that even large companies are still using small (7.6 cm or 3-inch) tube furnaces to grow the h-BN for their prototypes^45^. We also confirm that getting the microchips finalized and etch the passivation film before transferring the h-BN works well.

### Device characterization

The morphology of the devices was investigated by an optical microscope (DM 4000M, Leica), AFM (Dimension Icon, Bruker) and TEM (Titan Themis, FEI). The thin lamellae for TEM inspection were prepared using a scanning electron microscope provided with focused ion beam (Helios G4 UX, Thermo Fisher Scientific). The electrical characterization was performed by using two probe stations (both M150, Cascade) connected to different semiconductor parameter analysers: a Keithley 4200 and a Keysight B1500A. All the *IV* curves under DC voltages were collected using the Keithley 4200 in the ramped voltage sweep mode, for which three source-measure units are needed for drain, source and gate. Also, all the *IV* curves under pulse mode were collected by Keysight B1500A with two Waveform Generator/Fast Measurement Units connected to the drain and source. An Agilent E3631A DC Power Supply was used to apply constant voltage stress on the transistor gate as gate voltage for pulse measurement. All the endurance plots were collected using the recommended method described in ref. ^42^.

### SNN simulation

The SNN architecture^20^ has been developed using Brian2 (ref. ^46^), an SNN simulator written in Python. The learning process is based on the empirical measurement of STDP made in the 1T1M cells combining an Au–Ti–h-BN–W memristor on a CMOS transistor. We considered the variability of the network by running a Monte Carlo engine. We benchmarked the accuracy of the SNN during image classification^20^ of the MNIST dataset of handwritten digits^21^ under an unsupervised learning scheme. We propose a circuit-level model for the neuron–synapse–neuron system, as well as its implementation in SPICE. A detailed description of the SNN and its performance is given in Supplementary Note 3.

## Online content

Any methods, additional references, Nature Portfolio reporting summaries, source data, extended data, supplementary information, acknowledgements, peer review information; details of author contributions and competing interests; and statements of data and code availability are available at 10.1038/s41586-023-05973-1.

## Supplementary information

Supplementary Information This file contains Supplementary Figs. 1–14, Tables 1–5 and References.

## Data Availability

The data needed to evaluate the conclusions in this work are publicly available online at 10.5281/zenodo.7607096. The datasets that we used for benchmarking are publicly available in ref. ^21^. The training methods are provided in ref. ^20^.

## Code availability

The simulator Brian2 used here is publicly available in ref. ^46^. The codes used for the simulations described in Methods are publicly available online at 10.5281/zenodo.7607096.
