# Supplementary material for: Hybrid 2D–CMOS microchips for memristive applications
Source: Nature. 2023 Mar 27;618(7963):57–62. doi: 10.1038/s41586-023-05973-1 (PMC10232361; doi:10.1038/s41586-023-05973-1)
Supplement: Supplementary file 1 — This file contains Supplementary Figs. 1–14, Tables 1–5 and References. [file 41586_2023_5973_MOESM1_ESM.pdf]

nature portfolio

https://doi.org/10.1038/s41586-023-05973-1

Supplementary information

Hybrid 2D–CMOS microchips for memristive applications

In the format provided by the authors and unedited

Hybrid 2D/CMOS microchips for memristive applications

Kaichen Zhu[1]†, Sebastian Pazos[1]†, Fernando Aguirre[1], Yaqing Shen[1], Yue Yuan[1], Wenwen Zheng[1], Osamah Alharbi[1], Marco A. Villena[1], Bin Fang[1], Xinyi Li[2], Alessandro Milozzi[3], Matteo Farronato[3], Miguel Muñoz-Rojo[4-5], Tao Wang[6], Ren Li[7], Hossein Fariborzi[7], Juan B. Roldan[8], Guenther Benstetter[9], Xixiang Zhang[1], Husam Alshareef[1], Tibor Grasser[10], Huaqiang Wu[2], Daniele Ielmini[3], Mario Lanza[1]*

[1] Materials Science and Engineering Program, Physical Science and Engineering Division, King Abdullah University of Science and Technology (KAUST), Thuwal 23955-6900, Saudi Arabia
[2] Institute of Microelectronics, Tsinghua University, 100084, Beijing, China
[3] Dipartimento di Elettronica, Informazione e Bioingegneria, Politecnico di Milano, Piazza L. da Vinci 32, 20133 Milano, Italy
[4] Department of Thermal and Fluid Engineering, Faculty of Engineering Technology, University of Twente, Enschede, Netherlands
[5] Instituto de Micro y Nanotecnología, IMN-CNM, CSIC (CEI UAM+CSIC), Madrid, Spain.
[6] Institute of Functional Nano & Soft Materials, Collaborative Innovation Center of Suzhou Nanoscience and Technology, Soochow University, 199 Ren-Ai Road, Suzhou 215123, China
[7] Computer, Electrical and Mathematical Sciences and Engineering Division, King Abdullah University of Science and Technology, Thuwal, 23955-6900, Saudi Arabia
[8] Departamento de Electrónica y Tecnología de Computadores, Facultad de Ciencias, Universidad de Granada, Avenida Fuentenueva s/n, 18071 Granada, Spain
[9] Department of Electrical Engineering and Media Technology, Deggendorf Institute of Technology, Dieter-Görlitz-Platz 1, 94469 Deggendorf, Germany
[10] Institute for Microelectronics, TU Wien, Gusshausstrasse 27-29, 1040 Vienna, Austria

† These authors contributed equally
* Corresponding author email: mario.lanza@kaust.edu.sa

Table of contents

Page

Supplementary Figure 1 | Internal connections of the crossbar array 3
Supplementary Figure 2 | Devices and circuits in the microchip 4
Supplementary Figure 3 | Morphological characterization of tungsten vias of the fourth metallization layer 5
Supplementary Figure 4 | Wafer processing for cross-sectional TEM inspection 6
Supplementary Figure 5 | Size and chemical composition of the CMOS transistors 7
Supplementary Figure 6 | Standalone Au/Ti/h-BN/W structures that show bipolar RS 8
Supplementary Note 1: switching mechanism of the 1T1M cells 9
Supplementary Figure 7 | Resistive switching in atomic-layer-deposited TiO_2 10
Supplementary Figure 8 | Unstable non-volatile RS in an 1T1M cell with Au/Ti/h-BN/W memristor biased at V_G=1.5V 10
Supplementary Figure 9 | Morphological analysis 11
Supplementary Note 2: Endurance benchmarking.. 12
Supplementary Figure 10 | Importance of device size when benchmarking the endurance of RS devices.. 13
Supplementary Figure 11 | The two methods to characterize endurance 14
Supplementary Table 1 | Studies that report high endurance in small memristors with sizes below 1 µm^2 14
Supplementary Table 2 | Studies that claimed high endurance in large memristors with sizes above 1 µm^2 (independently on the characterization method used) 15
Supplementary Table 3 | Studies that claimed high endurance in small memristors with sizes below 1 µm^2 presenting few R_{HRS} and R_{LRS} data points 16
Supplementary Figure 12 | Hierarchy of electronic memories 17
Supplementary Note 3: Spike-timing dependence plasticity for spiking neural networks 18
Supplementary Figure 13 | Fitting of the STDP characteristic 19
Supplementary Figure 14 | Evolution of the synaptic connections between the input and excitatory layers during training for the case of 100 excitatory/inhibitory neurons, for different Monte-Carlo runs 20
Supplementary Table 4 | Classification of the MNIST dataset using Spiking Neural Networks 21
Supplementary Table 5 | List of articles from TSMC, Intel and Imec in the field of 2D materials that employ Au electrodes.. 22
Supplementary References.. 23

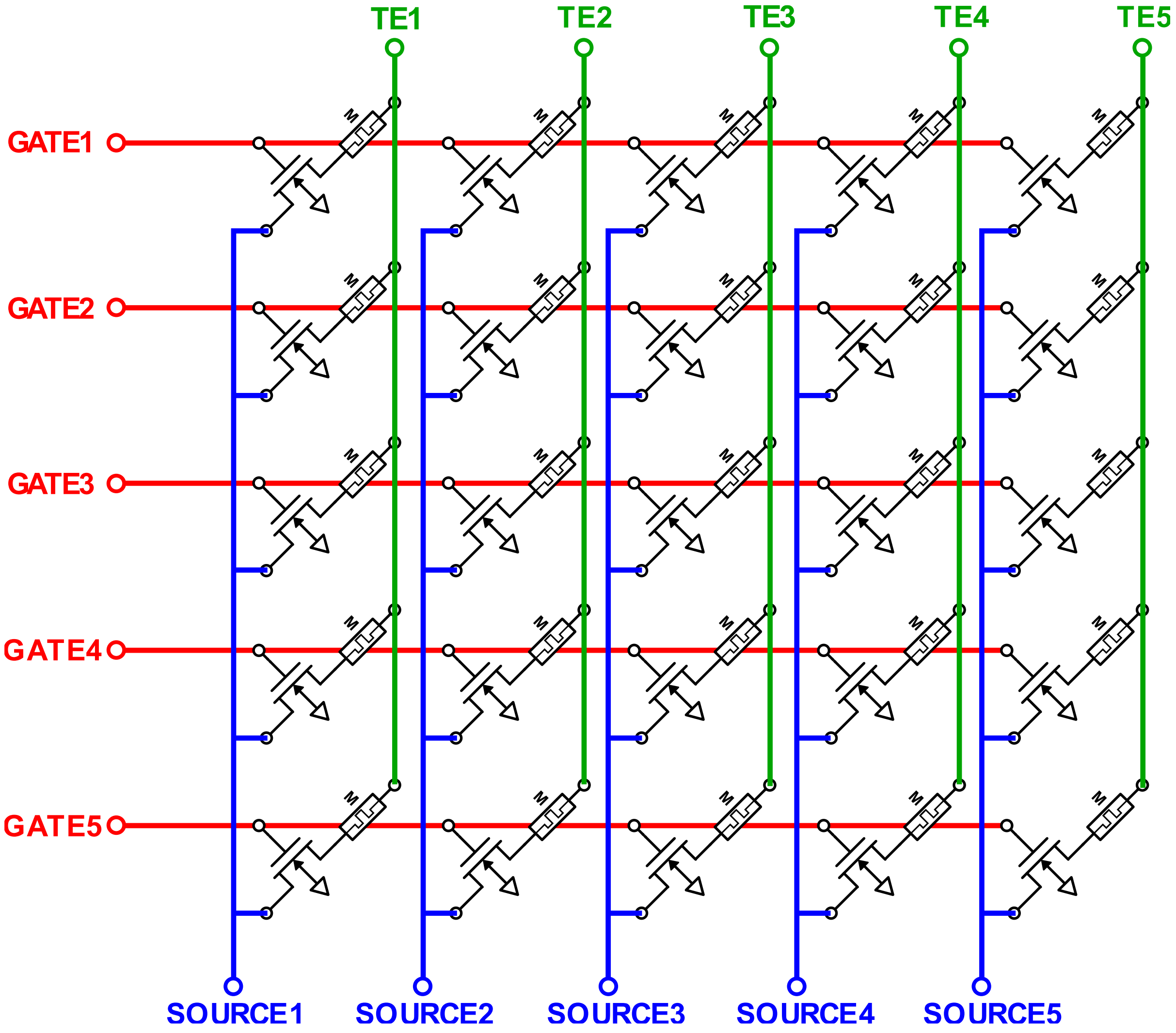

Supplementary Figure 1 | Internal connections of the crossbar array. Electrical schematic indicating the internal connections within the 5×5 crossbar arrays of 1T1M cells.

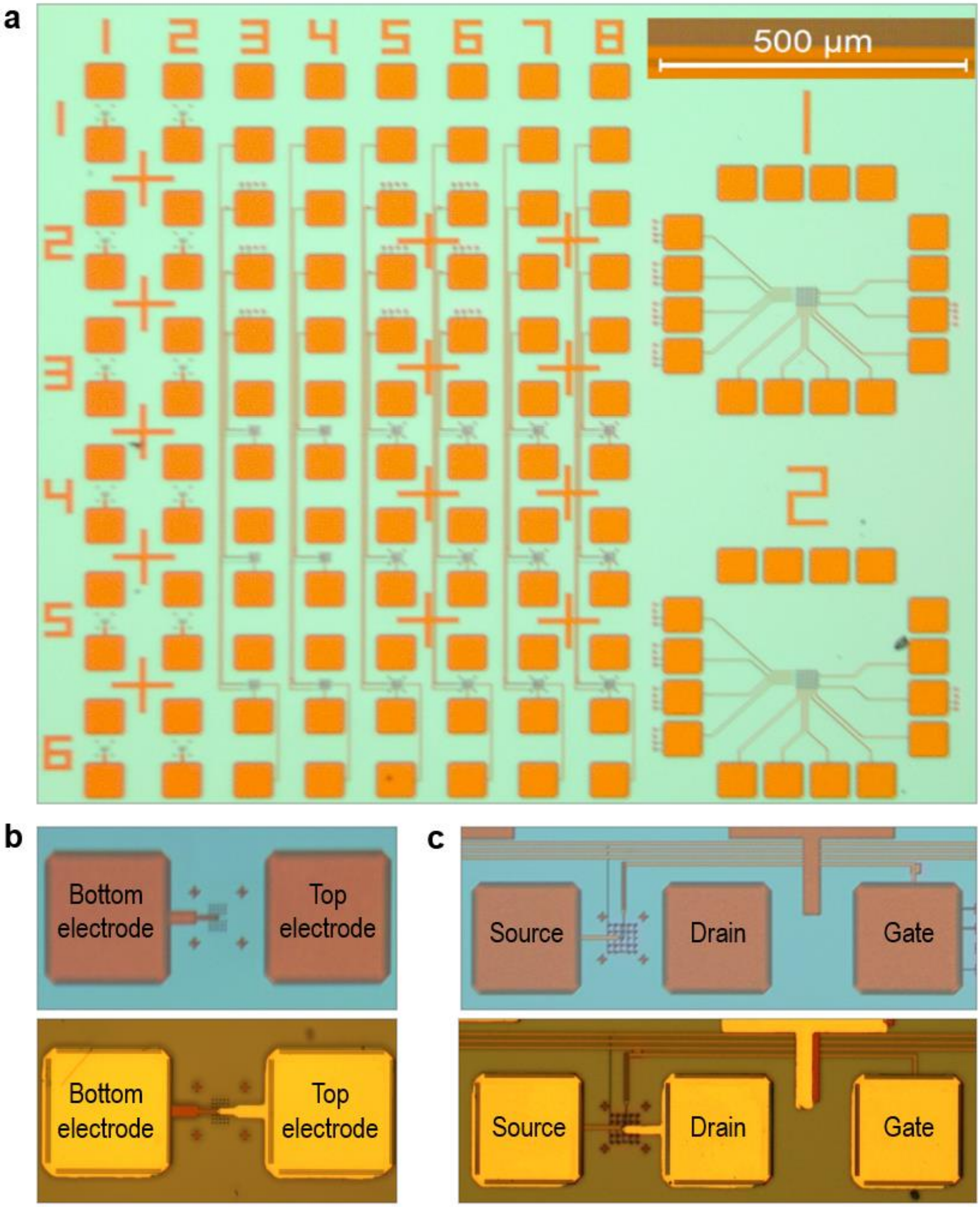

Supplementary Figure 2 | Devices and circuits in the microchip. a, Optical microscope image of the part of the microchip measured in this study, including standalone memristors (columns 1-2), standalone transistors (columns 3-8) and 5×5 crossbar arrays of 1T1M cells (right part), as-received from the foundry. **b**-**c**, Zoom-in optical microscope images of standalone h-BN memristor (**b**) and standalone transistor (**c**) before (up) and after (down) fabrication.

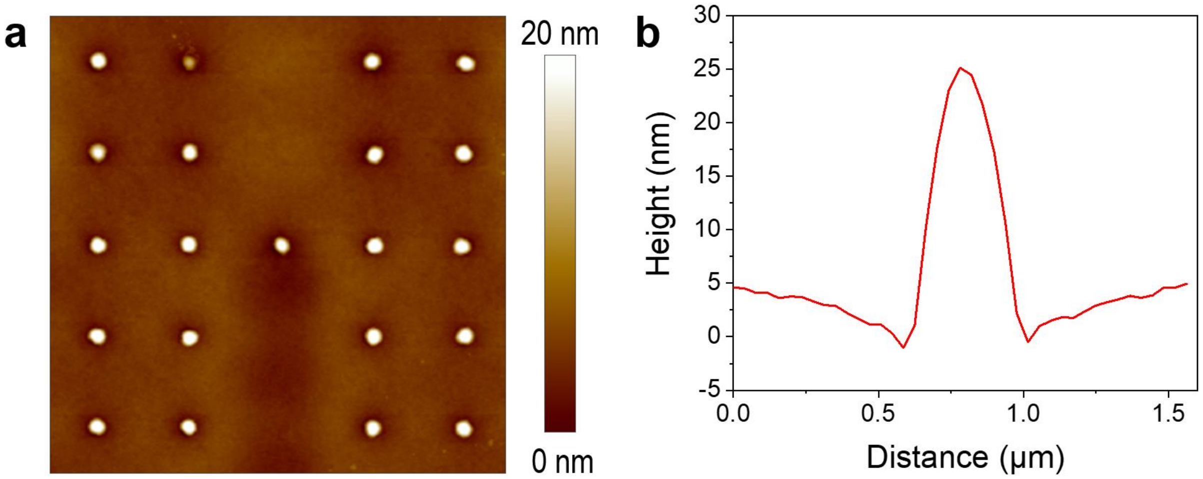

Supplementary Figure 3 | Morphological characterization of tungsten vias of the fourth metallization layer. a, Topographic AFM image of the surface of the silicon microchip (before transferring the h-BN sheet), in which multiple tungsten vias can be observed. The size of the image is 10 μm × 10 μm. This region corresponds to that of a single memristor (center) surrounded by many disconnected vias (often referred to as "dummy" vias). **b**, Cross-sectional view of a metallic via shown in the center of panel **a**.

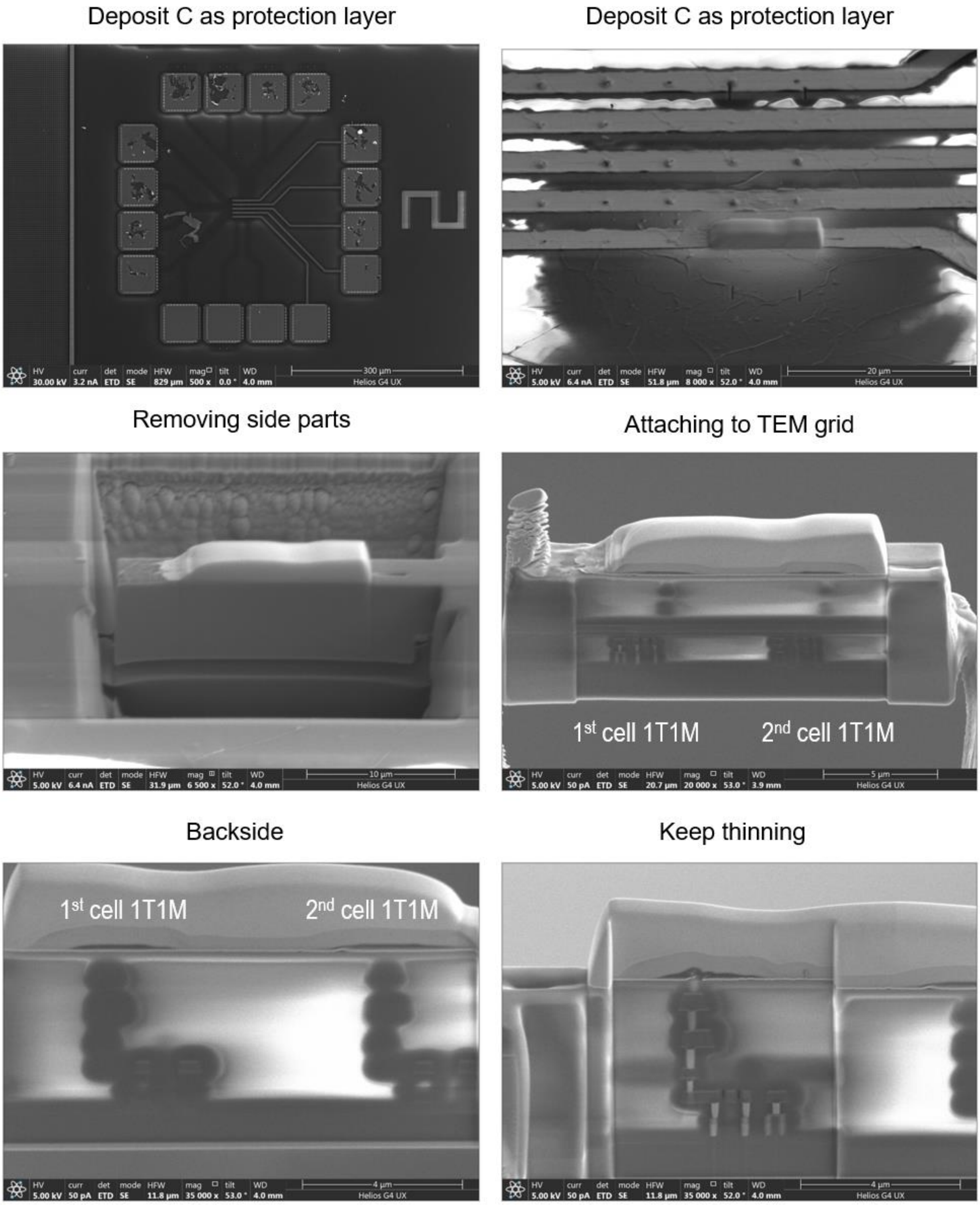

Supplementary Figure 4 | Wafer processing for cross-sectional TEM inspection. SEM images of the wafer at different stages of the focused ion beam process that exposed the transistors and vias. The transistors only appeared at the end of the process, i.e., when the lamellas were very thin, due to their small size.

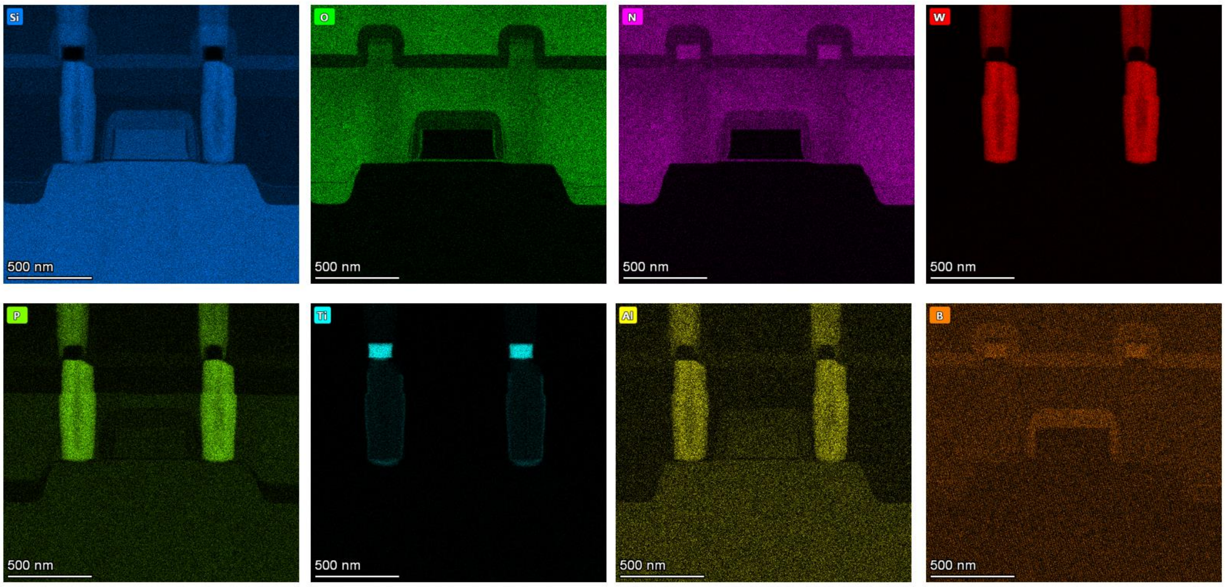

Supplementary Figure 5 | Size and chemical composition of the CMOS transistors. Cross-sectional energy dispersive X-Ray spectroscopy maps for different elements (Si, O, N, W, P, Ti, Al and B) in the transistors used in this investigation. The length of the channel is 500 nm.

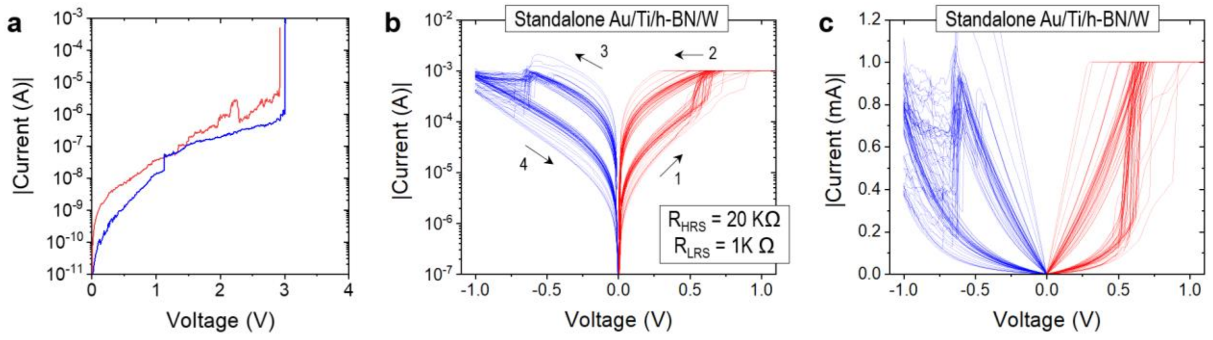

Supplementary Figure 6 | Standalone Au/Ti/h-BN/W structures that show bipolar RS. Current versus voltage plots measured in two different standalone Au/Ti/h-BN/W structures showing a forming voltage <3V. This behaviour has been observed in only ~10% of devices analysed, the other ~90% show no dielectric breakdown and erratic pre-breakdown current fluctuations (see Figure 2a in the main text). **b**, Current versus voltage plots measured in a standalone Au/Ti/h-BN/W structure (the one in red in panel **a**) showing non-volatile bipolar RS. The low values of the resistance in high resistive state (R_{HRS}=20KΩ) and low resistive state (R_{LRS}=1KΩ), the linearity of the currents in LRS, and the sharp set and reset transitions (at 0.59±0.04V and -0.66±0.04V, respectively) clearly indicate that the RS is filamentary. **c**, Data in panel **b** plotted in linear scale, showing that the currents in LRS are linear.

Supplementary Note 1: switching mechanism of the 1T1M cells

Previous stuies on h-BN based memristors have reported filamentary RS[1-10]. In none of those studies a series transistor was used. By combining multiple experiments, we conclude that the non-volatile bipolar RS observed in the 1T1M cells with ~0.053µm^2 memristors (Figure 2c of the main text) is non-filamentary, and related of ionic exchange at the interfaces between the h-BN and the electrodes when the h-BN is softly degraded (something that can only be done with the series CMOS transistor).

First, if we repeat the fabrication process but without transferring h-BN, the resulting Au/Ti/W, Au/W and Ag/W nanojunctions (with and without series CMOS transistor) don't exhibit RS. The Au/W and Ag/W nanojunctions exhibit ohmic conduction, and the Au/Ti/W nanojunctions exhibit slightly lower current due to the higher resistance of the Ti layer (which could absorb some oxygen, as shown in Extended Data Figure 3). In fact, atomic-layer-deposited 2-nm-thick TiO_2 also exhibits non-filamentary non-volatile bipolar RS (Supplementary Figure 7). However, atomic-layer-deposited 2-nm-thick TiO_2 is much more insulating than our O-rich Ti interfacial film, which is not responsible for the switching in our 1T1M cells because, as mentioned: i) devices with Au/Ti electrodes and without h-BN do not switch, and ii) devices without Au/Ti electrodes and with h-BN switch well (see Figure 2g of the main text). Hence, the non-volatile bipolar RS is related to the h-BN stack.

Second, our h-BN/CMOS based 1T1M cells biased at V_G=1.1V exhibit: i) very high R_{LRS}, ii) non-linear currents in both states, and iii) very progressive state transitions. These are very strong indications and widely-accepted evidences of non-filamentary RS, as explained in multiple seminal articles[11-12].

Third, when V_G is increased to 1.5V the currents in LRS become linear, as shown in the Supplementary Figure 8; this further confirms that the RS at V_G=1.1V is non-filamentary. Note that this behaviour is not stable and erratic transitions between filamentary and non-filamentary RS are detected.

And fourth, we have also conducted cross-sectional TEM coupled with EELS and EDX in our ~0.053µm^2 memristors (see Supplementary Figure 9a). We do see a partial degradation of the device, i.e. some black particles partially penetrate in the insulating h-BN. However, the size of these particles are very small compared to the filaments observed in 5µm^2 devices (see Supplementary Figure 9b), and they appear to be discontinuous. Moreover, we don't detect metal penetration via EELS/EDS.

For all these reasons, we cannot claim that the RS is filamentary. In our opinion, this is non-filamentary switching produced by the partial degradation of the h-BN consistent with the activation process detected (see blue lines in Figure 2c and Extended Data Figure 4).

Additional consideration

In memristors made of amorphous metal-oxides, the filamentary or non-filamentary nature of the RS could be also deduced by measuring devices with diffent areas. In non-filamentary devices the currents driven in LRS and HRS depend on the size, but in filamentary devices the currents driven in LRS do not depend on the size[11-12]. However, in polycrystalline metal-oxides this experiment may not be conclusive because, when the size of the memristor is smaller than the grain size (i.e., the device does not contain any grain boundary), a sharp change in the electrical properties could be observed.

In our case, the CVD h-BN is polycrystalline, i.e., it includes 2D layered regions that are electrically very insulating and clusters of defects that are more conucting —these clusters of defects may be related to grain boundaries or just lattice distortions that propagate from one layer to another[7-8]. This means that the electrical properties of a large device (>10µm^2) including one/few cluster/s of defects could be completely different to those of a small device (<0.1µm^2) made entirely of 2D layered h-BN. To confirm this hypothesis, we have fabricated h-BN memristors with different sizes: ~0.053µm^2, 0.5625µm^2, and 5µm^2. What we observe is: i) The 0.5625µm^2 and 5µm^2 devices show clear dielectric breakdown followed by filamentary non-volatile bipolar RS for tens/hundreds of cycles. The filamentary nature of the RS in these devices is confirmed by the sharp set/reset transitions, the very low value of R_{LRS}, and the linear currents detected in LRS. Before the first breakdown, no stable RS is seen. And ii) the ~0.053µm^2 devices exhibit stable non-volatile RS with: i) very high R_{LRS}, ii) non-linear currents, and iii) very progressive state transitions, as shown in the manuscript. As mentioned, these are very strong indications and widely-accepted evidences of non-filamentary RS[1-5].

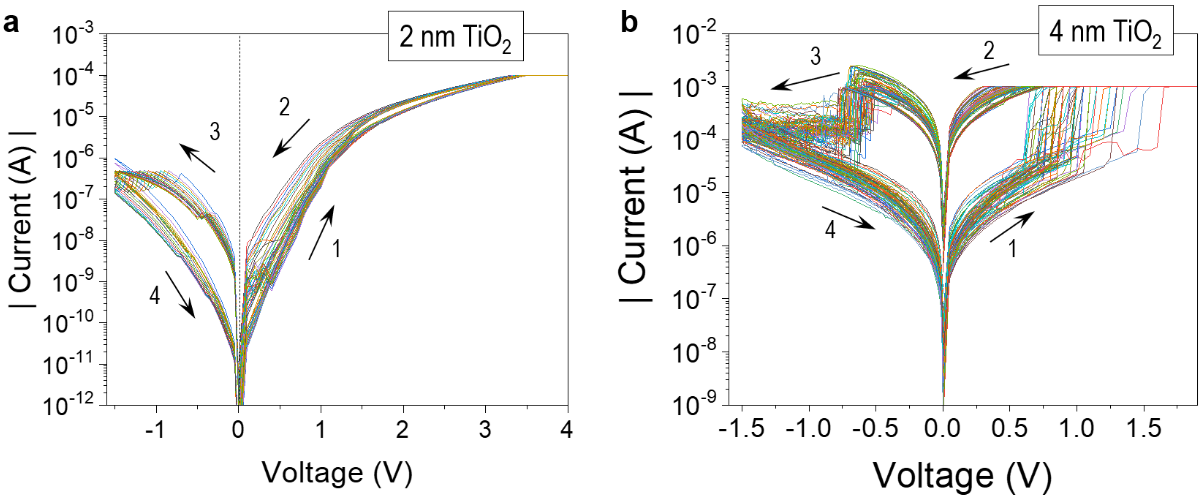

Supplementary Figure 7 | Resistive switching in atomic-layer-deposited TiO_2. Current versus voltage curves measured in TiO_2-based memristors with different thicknesses and top Au electrode. The bipolar RS in 4nm-thick TiO_2 (and thicker, see reference 13) shows characteristic filamentary non-volatile bipolar RS, but 2-nm-thick TiO_2 shows non-filamentary. However, this film is not responsible for the switching in our 1T1M cells because: i) devices with Au/Ti electrodes and without h-BN do not switch, and ii) devices with Ag and Au electrodes (i.e., without Ti) switch well (see Figure 2g of the main text).

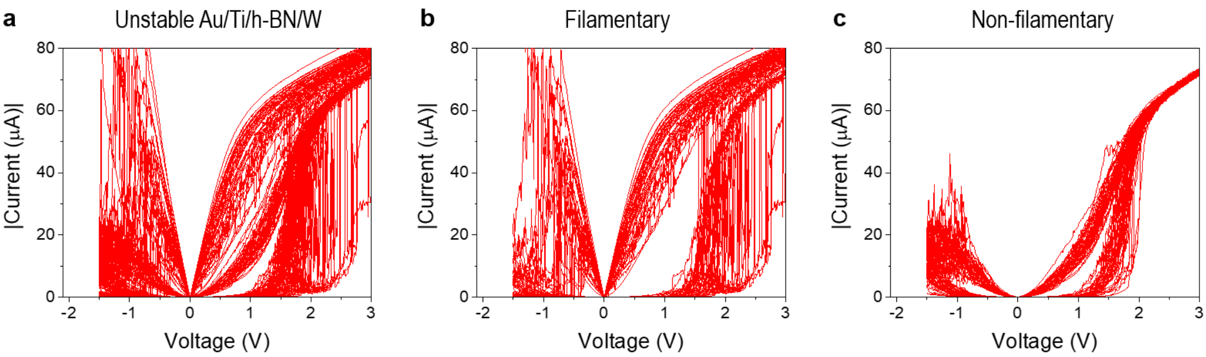

Supplementary Figure 8 | Unstable non-volatile bipolar RS in an 1T1M cell with Au/Ti/h-BN/W memristor biased at V_G=1.5V. Current versus voltage curves measured in an Au/Ti/h-BN/W device that shows high cycle-to-cycle variability. By splitting the curves in two groups we can clearly see that this variability is related to stochastic changes of the RS mechanism from filamentary (linear R_{LRS}, panel **b**) to non-filamentary (non-linear R_{LRS}, panel **c**), and vice versa. This happens because the value of R_{LRS} is not high enough to remain in the non-filamentary regime and not low enough to remain in the filamentary regime.

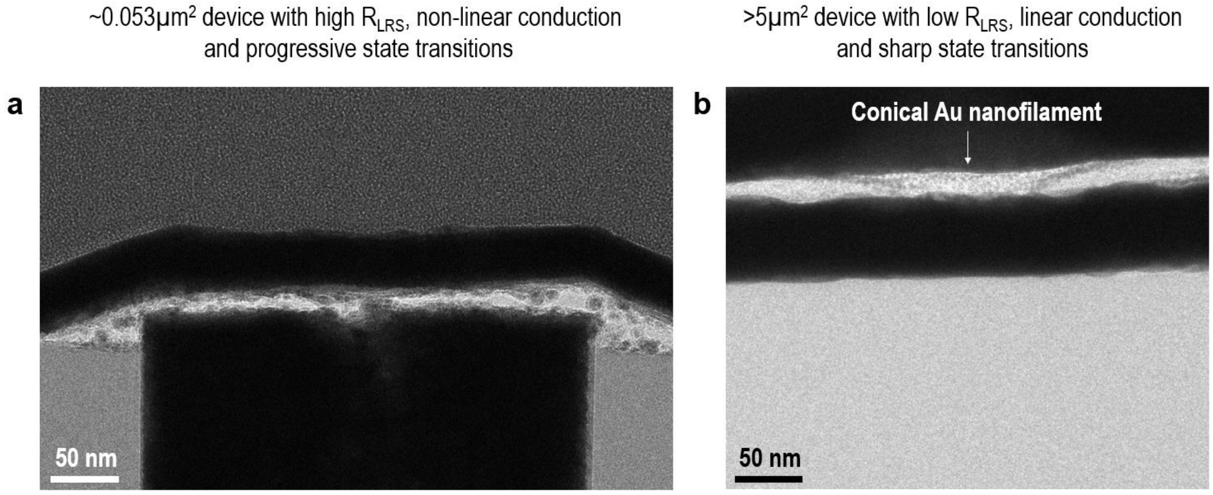

Supplementary Figure 9 | Morphological analysis. a-b, Cross sectional TEM image of two stressed CVD h-BN memristors with small and large size (respectively). The large devices clearly show the formation of wide conical nanofilaments (panel **b**), while the small devices show partial degradation with just few small particles displacement. These particles do not seem to be forming an effective filament but it seems to be partially broken (having nanogaps). These devices do not show metal migration in the EELS/EDX maps.

Supplementary Note 2: Endurance benchmarking

When benchmarking the endurance of memristive devices, one has to be very careful with two issues:

1 – Most articles that claimed high endurance (>10^6 cycles) studied large devices (>1μm^2). The fact that one large device exhibits one kind of RS does not mean that a small device (<0.1μm^2) made of the same materials will exhibit similar RS. This is evidenced by the fact that multiple articles have claimed high endurance up to ~10^{12} cycles in large devices (many of them even without using a series transistor to limit the current overshoot), but companies like Fujitsu and Intel/Micron qualify their commercial (small) metal-oxide and phase-change memories for only 0.5 million and 10 million cycles (respectively), despite using professional testing vehicles and selector elements[14-15].

It is widely known that in most memristive devices the forming voltage remarkably increases (statistically) in smaller devices[9,16-19], due to the lower probability to find native defects that trigger the dielectric breakdown (see Supplementary Figure 10a-d). Materials with lower density of defects tend to show no/bad RS. For example, SiO_2 produced by thermal oxidation and mechanically exfoliated h-BN (very low density of native defects) do not show stable RS, while SiO_2 produced by sputtering and CVD-grown h-BN (high density of native defects) exhibit stable RS[8,20]. In general, higher breakdown voltages in materials with lower amount of native defects will produce wider filaments (see Supplementary Figure 10e-h); moreover, the higher electrical field will contribute to generate electromigration and self-accelerated avalanche currents, possibly resulting in filaments with different chemical composition (see the orange and red balls in the filaments of Supplementary Figure 10h). Larger filaments are good to enhance the state retention time, but at the same they reduce the switching endurance because they tend to get stuck in LRS (this is an inherent trade-off in many memristive devices[21]).

Hence, when benchmarking the endurance of a memristor, the concept of device size is very important. It is misleading to measure an endurance of 10 billion cycles in a memristor with size of >10μm^2 and claim that it could be used as electronic memory, because electronic memories require a high integration density and high endurance (simultaneously). Such endurance must be confirmed in smaller devices with an integration density suitable for memory applications (<0.1μm^2). Supplementaary Figure 10i-j show the concept idea of our devices: the high endurance comes from the fact that no complete dielectric breakdown is triggered.

2 – Many plots used to claim high endurance present very few data, i.e., read R_{LRS} and R_{HRS} just one/few times per decade for only one device (see Supplementary Figure 11a). Such type of claim is extremely weak. We note that multiple claims of high endurance in novel nanomaterials have never been reproduced by other groups. Recently, a group of experienced researchers in the field of RS published an article exposing this bad practice[22], i.e., explaining why the characterization method shown in Supplementary Figure 11a is unreliable, and urging the community to employ a reliable method, which consists on measuring R_{LRS} and R_{HRS} in each cycle for few devices (see Supplementary Figure 11b).

For these reasons, when benchmarking the endurance of our ~0.053μm^2 h-BN memristors, we only compare with articles that report high endurance in memristors with sizes <1μm^2 and that present an abundant number of data points for R_{HRS} and R_{LRS}. We selected 1μm^2 as threshold because this device size starts to be attractive for on-chip memory applications. Note that, due to the limited resolution of the plots, we cannot be 100% sure if an endurance plot in an article read R_{HRS} and R_{LRS} in every cycle, unless the authors explicitly mention it in the text —we fully support data availability policies from some publishers. Therefore, we will select those studies in which the number of data points is so abundant that they cannot be counted (i.e., the number of data points is indistinguishable).

We have made a deep literature review and we only found 4 studies that demonstrated (presenting abundant data on R_{HRS} and R_{LRS}) high endurance in small (<1μm^2) memristors, as shown in Supplementary Table 1. In conclusion, the ~0.053μm^2 h-BN memristive devices presented in our study achieved a high endurance, which is competitive compared with memristors made of other much more mature materials, such as metal-oxides, phase-change materials and amorphous silicon.

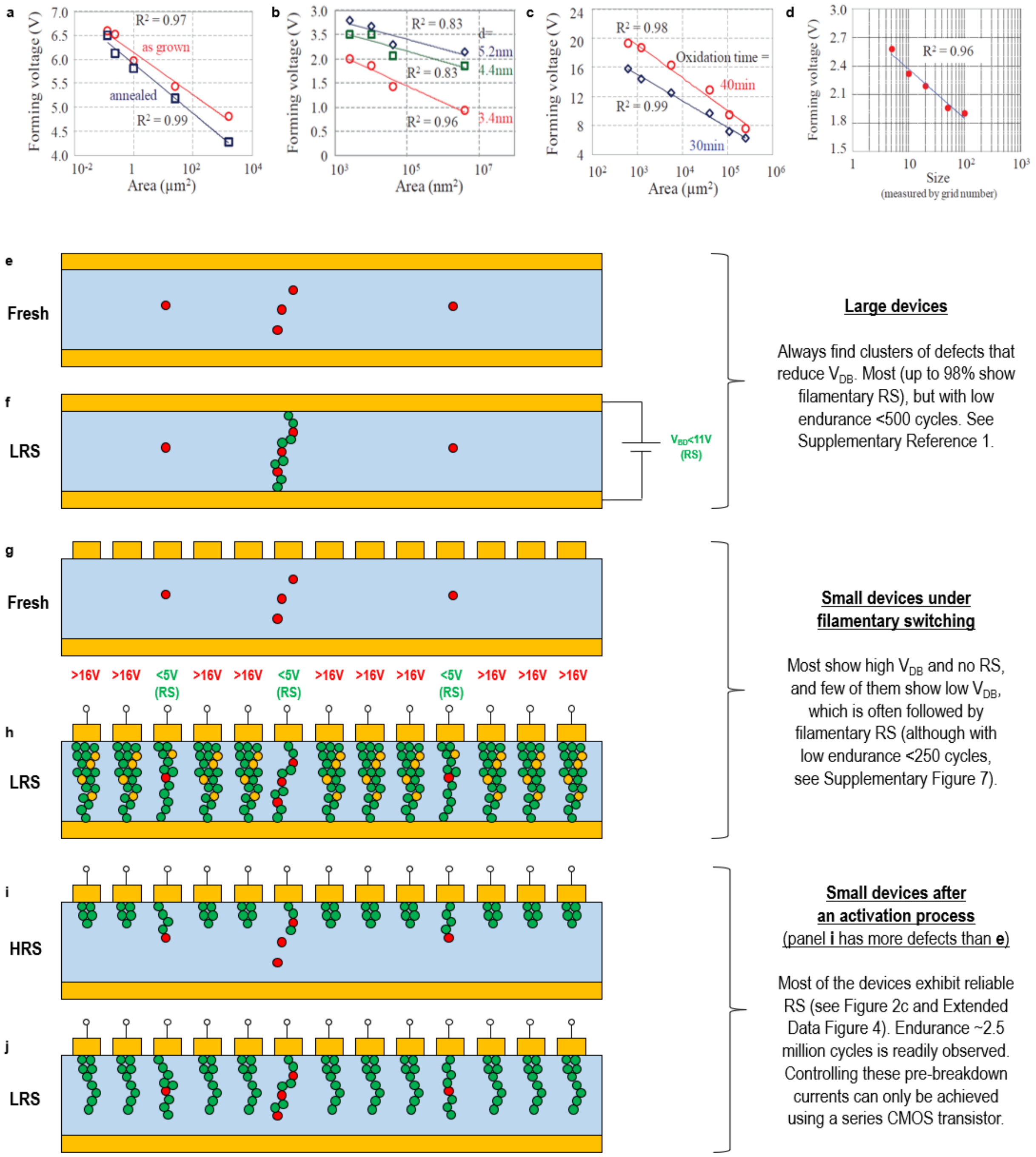

Supplementary Figure 10 | Importance of device size when benchmarking the endurance of RS devices. **a**, Forming voltage versus device area of resistive random access memories (RRAM) with 20nm thick annealed and as-grown HfO_X. **b**, Forming voltage versus device area of HfO_X RRAM for oxide thickness of 3.4nm, 4.4nm, and 5.2nm. **c**, Forming voltage vs. device area of CuO_X RRAM with oxidation time of 30min and 40min. **d**, The simulated median forming voltage versus device size. **a-d**, are reproduced with permission from reference 9, copyright IEEE 2013. **b-g**, Schematic representation and explanation of the switching in memristors with different sizes, as described in the text on the right side).

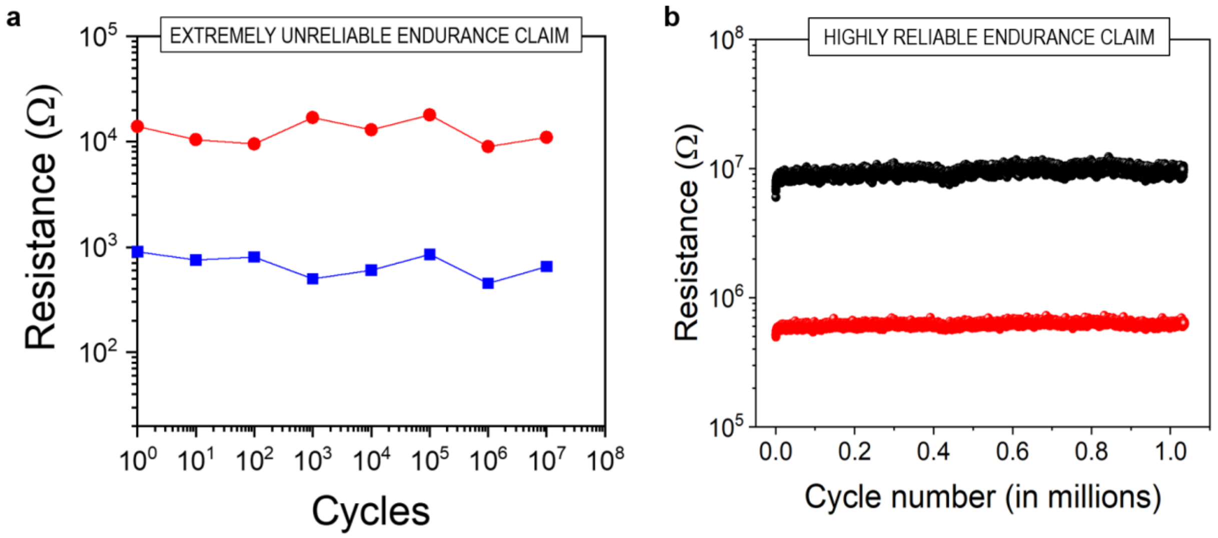

Supplementary Figure 11 | The two methods to characterize endurance. a, Unreliable endurance claim by reading R_{HRS} and R_{LRS} few times (one or few per decade). **b**, Highly-reliable endurance claim based on a plot that presents the value of R_{HRS} and R_{LRS} in each cycle.

Supplementary Table 1 | Studies that report high endurance in small memristors with sizes below 1 µm^2. These studies used the correct characterization method (one data point per cycle, which is the only one accepted by most of the experts in this field, see reference 22).

Reference (group)	Structure	RS mode	Device area	Endurance claimed	Data points presented	Reliability of the claim
Our work	Au/Ti/h-BN/W	Bipolar	~0.053 µm^2	5 × 10^6	indistinguishable	high
	Au/h-BN/W	Bipolar	~0.053 µm^2	2.5 × 10^6	indistinguishable	high
23 (NTHU)	TiN/TiO_x/HfO_x/TiN	Bipolar	0.12 µm^2	>10^6	indistinguishable	high
24 (CEA-LETI)	Ag/10%Sb-GeS_2/W	Bipolar	0.031 µm^2	>10^5	indistinguishable	high
25 (Wei Lu)	Ag/a-Si/poly-Si	Threshold	0.01 µm^2	>10^8	indistinguishable	medium
26 (Panasonic)	Pt/TaO_x/Pt	Bipolar	0.25 µm^2	>10^9	indistinguishable	medium

Supplementary Tables 2-3 show all the studies claiming high endurance that we discarded, either because they employ a large device size (>1µm^2) or because they presented very few data points in their endurance plots (or both reasons).

Supplementary Table 2 | Studies that claimed high endurance in large memristors with sizes above 1 μm² (independently on the characterization method used). Weaknesses highlighted in red.

Ref	Structure	Regimes	Device area	Endurance claimed	Data points presented	Reliability of the claim
27	$Ta/TaO_x/Pt$	Bipolar	7,853 μm²	$>10^{10}$	indistinguishable	high
28	$Pt/Ta/ZrO_2/Pt$	Bipolar	49 μm²	$> 10^6$	indistinguishable	high
29	$Pt/Ta/TaO_x$:Zr/Pt	Bipolar	4 μm²	2.9×10^{10}	indistinguishable	medium
30	$Ta/TaO_x/Pt$	Bipolar	3.14 μm²	10^6	indistinguishable	medium
31	Au/BFO/BFTO/Pt	Bipolar	45,000 μm²	$>2 \times 10^5$	indistinguishable	medium
32	Au/Co-doped $BaTiO_3$ (BTCO)/Pt	Unipolar	7,854 μm²	10^5	indistinguishable	medium
33	Pt/BST/SRO/STO	Bipolar	100 μm²	10^4	indistinguishable	medium
34	Al/PEDOT:PSS/PMMA/ITO	Bipolar	196,349 μm²	10^5	indistinguishable	medium
35	$Pt/TaO_x/Ta_2O_5/Pt$<br>$W/AlO/TaO_x$/Doped ZrO_x/Ru	Bipolar<br>Bipolar	900 μm²<br>900 μm²	$>10^{12}$<br>$>10^{11}$	~50<br>23	Low<br>low
36	$Ta/HfO_2/Pt$	Bipolar	100 μm²	1.2×10^{11}	~34	low
37	$Ti/HfO_2(1)/O_2$-$HfO_2(9)$/TiN	Bipolar	25 μm²	10^{10}	11	low
38	Al/PFCF-rGO/ITO	Bipolar	160,000 μm²	10^8	9	low
39	Ti/AlN/Pt	Bipolar	31,415 μm²	10^8	9	low
40	$ITO/HfO_x/ITO$	Bipolar	7,854 μm²	10^7	~150	low
41	Pt/SiO_2:Pt/Ta	Bipolar<br>Bipolar	25 μm²<br>1,963 μm²	(10 cycles)<br>3×10^7	~80	low
42	$G/MoS_{2-x}O_x/G$	Bipolar	1 μm²	$>2 \times 10^7$	~70	low
43	ITO/AlN/ITO	Bipolar	31,415 μm²	10^8	9	low
44	$TaN/ZrO_2/HfO_2$/TiN	Bipolar	17,671 μm²	10^7	~70	low
45	Pt/Zn:SiO_x/TiN	Bipolar	12.6 μm²	10^7	~70	low
46	Cu/ graphene/HfO_2/Pt	Bipolar	4 μm²	10^7	11	low
47	Graphene/PMMA:P3BT/Al/PET	Bipolar	230,000 μm²	10^7	15	low
48	Pt/TiO_2/TiN/Pt	Bipolar	400 μm²	2×10^6	24	low
49	Pt/Nb-doped $SrTiO_3$/Pt	Bipolar	900 μm²	$>10^6$	7	low
50	PtSi-coated AFM tips/HZO/LSMO/LAO	Bipolar	400 μm²	$>10^6$	13	low
51	Ag/GeSe/Si_3N_4/W	Bipolar	49,087 μm²	$>10^6$	11	low
52	Au/Pt/$Bi_{1-\delta}FeO_3$/ $SrRuO_3/SrTiO_3$	Bipolar	10,000 μm²	10^6	~55	low
53	Pt/ZnO/Pt	Bipolar	127,000 μm²	10^6	7	low
54	Pt/Ni:SiO_2/TiN	Bipolar	12.6 μm²	10^6	~54	low
55	Pd/Ag/HfO_x/Ag/Pd	Threshold<br>Threshold	0.01 μm²<br>25 μm²	-<br>$> 10^8$	-<br>indistinguishable	-<br>medium

Supplementary Table 3 | Studies that claimed high endurance in small memristors with sizes below 1 µm² presenting few R_{HRS} and R_{LRS} data points. Weaknesses highlighted in red.

Ref	Structure	Regimes	Device area	Endurance claimed	Data points presented	Reliability of the claim
56	$Ta/TaO_x/TiO_2/Ti$	Threshold	0.2 µm²	10^{13}	~21	low
57	$Pt/Ta_2O_{5-x}/TaO_{2-x}/Pt$	Bipolar	0.0009 µm²<br>0.25 µm²<br>900 µm²	-<br>10^{11}<br>10^{12}	-<br>~75<br>~75	low
58	TE/SLT/BE (1S)	Threshold	0.01 µm²	10^{11}	~60	low
59	$Ag/Ag_{33}Ge_{20}Se_{47}/Ag$	Bipolar	0.012 µm²	$>10^{10}$	4	low
60	$TiN/Hf/HfO_2/TiN$	Bipolar	0.0016 µm²	10^{10}	~31	low
	$TiN/Ti/HfO_2/TiN$	Bipolar	0.0016 µm²	10^{10}	~31	low
	$TiN/Ta/HfO_2/TiN$	Bipolar	0.0016 µm²	10^{6}	25	low
61	$TiN/Hf/HfO_2/TiN$	Bipolar	0.0016 µm²	10^{10}	~31	low
62	$Cu/HfO_2/Pt$ (1T1R)	Bipolar	0.12 µm²	$>10^{8}$	13	low
63	TiN/AsTeGeSiN/TiN/Pt	Threshold	0.25 µm²	10^{8}	25	low
64	$Cu/HfO_2/TiN/Ru$	Bipolar	0.2 µm²	10^{8}	8	low
65	TiN/RTO $WO_x/W/TiN$	Unipolar	0.0254 µm²	$>10^{7}$	~100	low
66	$Cu/Cu\text{-}Te/GdO_x/W$	Bipolar	0.0013 µm²	$>10^{7}$	~44	low
67	TiN/a-C:H/Pt	Bipolar	0.36 µm²	10^{7}	9	low
68	$TiN/Hf/HfO_2/TiN$	Bipolar	0.0001 µm²	10^{7}	~30	low
69	TiN/Hf/TaO/HfAlO/AlO/TiN	Bipolar	0.0016 µm²	10^{6}	~22	low
70	$TiN/Ti/HfO_x/TiN$	Bipolar	0.1296 to 3,600 µm²	10^{6}	~80	low
71	$TiN/WO_x/W$	Bipolar	0.0036 µm²	10^{6}	~70	low
72	noble metal/NiO/noble metal	Unipolar	0.21 µm²	10^{6}	~60	low
73	$Pt/ZrO_x/HfO_x/TiN$	Bipolar	0.002 µm²	10^{6}	7	low
74	Pt/Al/PCMO/Pt	Bipolar	0.002 µm²<br>2,500 µm²	10^{6}<br>10^{3}	~37	low
75	$Al/TiN/Cu/TiW/Al_2O_3/W$	Bipolar	0.0064 µm²	10^{6}	25	low

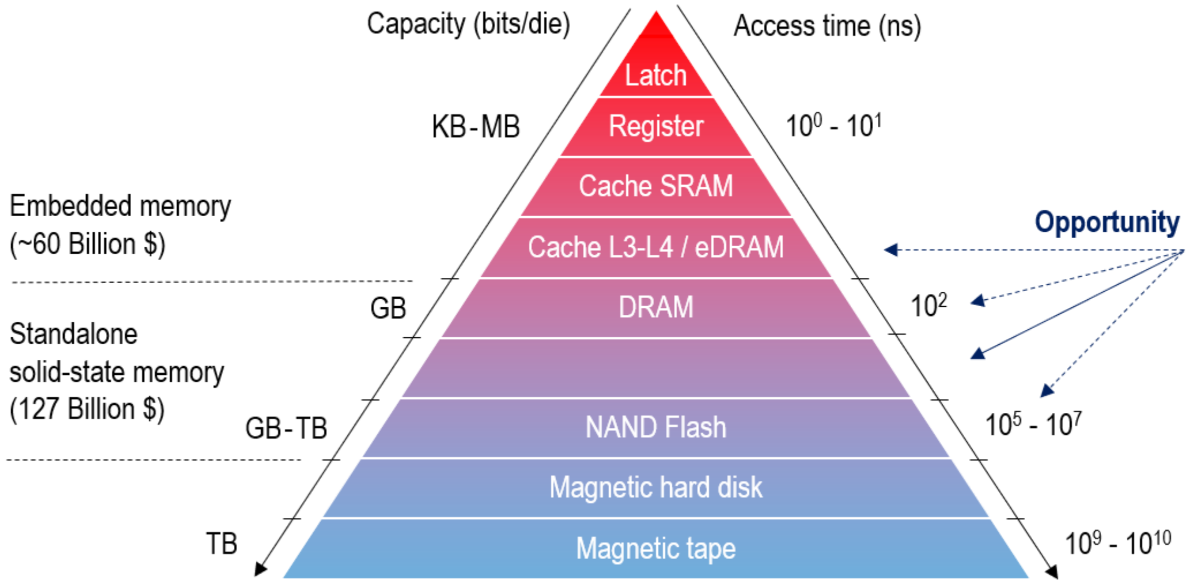

Supplementary Figure 12 | Hierarchy of electronic memories. Pyramidal representation of the capacity and access time of different types of electronic memories available in the market. The size of the market according to reference 76 is displayed on the left side of the pyramid. The gap between NAND Flash and DRAM (i.e., dynamic random access memory) could be a promising niche market for the hybrid h-BN/CMOS memories, given their performance.

Supplementary Note 3: Spike-timing dependence plasticity for spiking neural networks

1T1M cells with Au/Ti/h-BN/W memristors exhibit spike-timing dependent plasticity (STDP) when applying pulsed voltage stress (PVS) displaced in time (Δt) to the top (at time t_{post}) and bottom (at t_{pre}) electrodes of the Au/Ti/h-BN/W memristors. The device conductance variation (ΔG as a function of Δt, with Δt= t_{post}-t_{pre}) after each pair of pulses was measured with respect to the initial conductance ($G_{INITIAL}$) and the resulting STDP characteristic is presented in Supplementary Figure 13, showing an exponential trend instead a linear one as reported in previous cases[8, 82]. This non-volatile resistive switching performance is very attractive to construct electronic synapses for Spiking Neural Networks (SNNs)[83], which have become very attractive because they consume less energy than traditional deep neural networks[84]. Based on the measured STDP characteristic (which we fitted with a piecewise exponentially decaying model as shown in Supplementary Figure 14) we emulated the performance of a memristive SNN for classifiying[85] the images of the Modified National Institute of Standards and Technology (MNIST) dataset of handwritten digits[86] under an unsupervised learning scheme and proposed a circuit-level model for the neuron-synapse-neuron system.

The SNN architecture[85] is described in Figure 3a. This network has been developed using Brian2[87], an SNN simulator written in Python. Our SNN comprises 784 input neurons (one for each pixel in the dataset images), and 400 excitatory and inhibitory neurons (the SNN structure is then 784×400×400). The selected architecture allows lateral inhibition that leads the excitatory neurons to compete[85]. Neurons are modelled with the leaky integrate-and-fire (I&F) model[85, 88] and to prevent any of them from dominating the response pattern we employ an adaptive membrane threshold[89], meaning that the firing threshold is increased every time the neuron fires and otherwise it decays exponentially[90]. During training, the synaptic weight update due to a stimulation protocol with pairs of pre- and postsynaptic spikes can be calculated based on the STDP function[91, 92]. In this work, the online version of the STDP learning rule is employed in order to improve the simulation efficiency[93]. Further details regarding the STDP can be found in Ref. 82.

To study the impact of the device variability we considered a Monte-Carlo approach and repeated the training 50 times for each SNN, totalling 150 runs, each of them taking on ~20 hours. This approach implies that both the STDP characteristic and the initial value of the synapses changes from one Monte Carlo run to another, resulting in different conductance maps for each run (see Supplementary Figure 14). For each Monte-Carlo run, as the training progresses, the synapses connected to each of the excitatory neurons learn the general features of a given pattern. This is shown in Figure 3b, where each slice presents the 313,600 synapses arranged in 400 groups (20×20, i.e., the numeric patterns) of 784 synapses each (28×28, i.e., the pixels that form each numeric pattern); the synapses connect the input layer to the excitatory layer (green and red spheres in Figure 3a, respectively). The red square in Figure 3b indicates a group containing the 784 synapses that connect the input neurons to the first neuron of the excitatory layer. We trained the SNN with the complete MNIST training dataset and the accuracy was evaluated every 1000 images. The behaviour of the SNN is verified by the corresponding confusion matrix (see Figure 3c) and the evolution of the averaged classification accuracy during training is presented in Figure 3d. The best average accuracy reaches ~90%, which is a very high value considering the simplicity of the SNN, its similarity to biological neural networks (i.e., the learning rule employed is STDP), and the unsupervised training protocol (see Supplementary Table 4).

Aiming to implement a hardware-based SNN accelerator exploiting the capabilities of our h-BN/CMOS based 1T1M cells, we propose the CMOS circuit[94] shown in Figure 3e for emulating the electrical response of a leaky integrate and fire (leaky I&F) neuron, which is capable of accounting for the adaptative firing threshold and the refractory period after firing. The entire circuit is simulated in SPICE considering all the active devices (transistors) to be from a 180 nm commercially available CMOS process and its response is calculated while the Au/Ti/h-BN/W memristors acting as synapse is modelled with the quasi-static memdiode model of the memristor[95-96]. The correct response of the circuit is demonstrated by the presynaptic and postsynaptic traces and the evolution of the membrane potential, presented in Figure 3f-g, respectively.

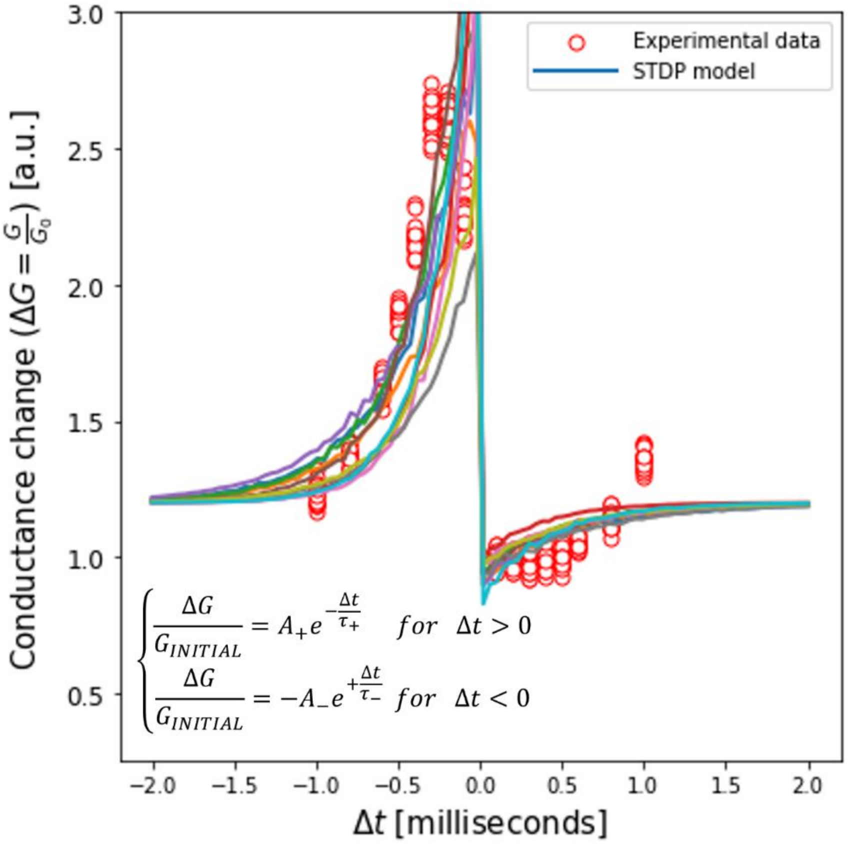

Supplementary Figure 13 | Fitting of the STDP characteristic. The asymmetric STDP characteristic of the CMOS/h-BN based 1T1M cell is fitted with the STDP model presented in the inset. The fitting parameters are: A_-=-0.03x10^{-6}, A_+=0.21x10^{-6}, τ_+=0.35 ms. and τ_-=0.5 ms. To account for the device-to-device variability, we included a 20% variability in the previously mentioned A_+ and τ_+ parameters.

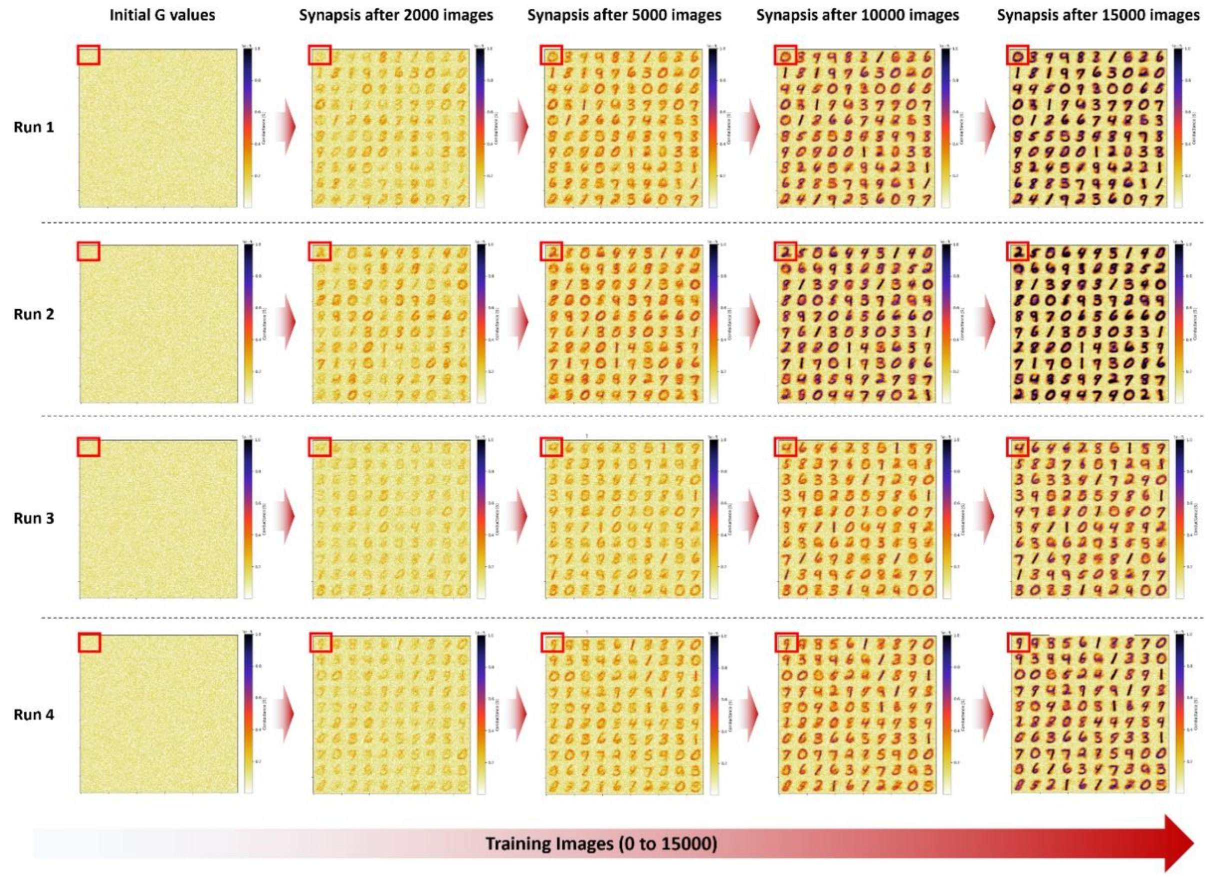

Supplementary Figure 14 | Evolution of the synaptic connections between the input and excitatory layers during training for the case of 100 excitatory/inhibitory neurons, for different Monte-Carlo runs. As different training examples from the MNIST dataset are presented to the SNN being investigated, the synapses (784) connected to a given neuron in the excitatory layer (100 neurons in total) learn the main features of a specific kind of pattern (i.e. a digit). For the sake of representation, the conductance values of the 784 synapses are represented in a 28×28 fashion, and then organized in a 10×10 matrix of this patterns. For brevity, only 4 from the 50 different Monte-Carlo runs are presented. Note that for each run, the matrix of learned patterns changes due to i) the random initial value of the synapses and ii) the variability of the STDP characteristic. i.e. in the first run the 1st excitatory neuron -upper left corner and highlighted by a red square- learns to recognize, for instance, the digit n° "0", then for the second run the same neuron learns to recognize the digit n° "2", for the third run digit n° "4" and for the fourth run digit n° "9"). Note that apart from different synaptic conductance maps, the device-to-device variability also produces changes in the conductance evolution (i.e. after 10,000 images, the conductance of the potentiated synapses in the map corresponding to the 4th Monte-Carlo run are sensibly lower than those from the 1st and 2nd Monte-Carlo runs. Note that for the sake of readability, in this case we have considered the case of 100 neurons, as for the 400 neurons case the patters are too small to be properly displayed in the figure.

Benchmarking of the SNN simulation

Supplementary Table 4 | Classification of the MNIST dataset using Spiking Neural Networks. The accuracy reported in this work is the highest among all other studies based on memristive synapses. Reference 100 uses a similar amount of synapses and reaches similar accuracy, but the complexity of each electronic synapse is much higher. References 85, 90 and 101 are included in this table, despite not employing any memristive synapse, to compare the accuracy of our network with that of purely theoretical

**Ref.**	**Synapse structure (complexity)**	**Memristor structure**	**Network**	**Learning method**	**Synapses**	**Accuracy**
97	1M (low)	$Al/Al_2O_3/TiO_{2-X}/Al$	SNN	STDP (unsupervised)	1960	61.5%
98	1M (low)	$Al/Al_2O_3/Nb_XO_Y/Au$	SNN	STDP (unsupervised)	7,840	65%
					15,680	70%
					39,200	77%
					78,400	82%
99	1T1M (medium)	$Ti/HfO_X/TiN$	SLP	Backpropagation (supervised)	11520	84%
100	6T2M (high)	PCM	RBM	STDP (unsupervised)	692,000	92%
**This Work**	**1T1M (medium)**	**Au/Ti/h-BN/W**	**SNN**	**STDP (unsupervised)**	**78,400**	**83.8%**
					**313,600**	**91.8%**
					**705,600**	**92.4%**
101	None (theoretical case)	None	SNN	Backpropagation (supervised)	2,392,800	98.6%
85	None (theoretical case)	None	SNN	STDP (unsupervised)	78,4000	82.9%
					313,600	87.0%
					1,254,400	91.9%
					5,017,600	95.0%
90	None (theoretical case)	None	SNN	STDP (unsupervised)	39,200	81%
					235,200	93.5%

Supplementary Table 5 | List of articles from TSMC, Intel and Imec in the field of 2D materials that employ Au electrodes. Note that the use of Au in the transistors is much worse than in memristors, as the transistors are in the FEOL and the memristors in the BEOL (see Extended Data Figure 10).

Ref.	Authorship	Device studied	Metal contact used
102	TSMC	MoS_2 transistor	Sb/Au
103	TSMC	MoS_2 transistor	Au
104	Intel	WS_2 transistor	Ti/Au
105	Intel	MoS_2 transistor	Au
106	Imec	WS_2 transistor	Ti/Au
107	Imec	MoS_2 transistor	TiN/Au

Supplementary References

[1] Chen, S. et al. Wafer-scale integration of two-dimensional materials in high-density memristive crossbar arrays for artificial neural networks. *Nat. Electron.* **3**, 638–645 (2020).

[2] Shen, Y. et al, Variability and yield in h-BN-based memristive circuits: the role of each type of defect. *Adv. Mater.* **33**, 2103656 (2021).

[3] Lin, W. et al. Oxygen-assisted synthesis of hBN films for resistive random access memories. *Appl. Phys. Lett.* **115**, 073101 (2019).

[4] Qian, K. et al. Hexagonal boron nitride thin film for flexible resistive memory applications. *Adv. Funct. Mater.* **26**, 2176–2184 (2016).

[5] Zhuang, P. et al. Nonpolar resistive switching of multilayer-hBN-based memories. *Adv. Electron. Mater.* **6**, 1900979 (2020).

[6] Wu, X. et al. Thinnest nonvolatile memory based on monolayer h-BN. *Adv. Mater.* 1806790 (2019).

[7] Pan, C. et al. Coexistence of grain-boundaries-assisted bipolar and threshold resistive switching in multilayer hexagonal boron nitride. *Adv. Funct. Mater.* **27**, 1604811 (2017).

[8] Shi, Y. et al. Electronic synapses made of layered two-dimensional materials. *Nat. Electron.* **1**, 458–465 (2018).

[9] Chen, A. Forming voltage scaling of resistive switching memories. *71st Device Research Conference.* 181–182 (2013).

[10] Cha, E. et al. Comprehensive scaling study of NbO2 insulator-metal-transition selector for crosspoint array application. *Appl. Phys. Lett.* **108**, 153502 (2016).

[11] Ielmini, D.; Waser, R., Resistive Switching: From Fundamentals of Nanoionic Redox Processes to Memristive Device Applications. John Wiley & Sons. Mannheim, 2016.

[12] Sawa. A, Resistive switching in transition metal oxides. *Mater. Today* **11**, 28–36 (2008).

[13] Acharyya, D. et al. A journey towards reliability improvement of TiO2 based Resistive Random Access Memory: A review. *Microelectron. Reliab.* **54**, 541–560 (2014).

[14] Website of Fujitsu. Non-volatile memory with very small sperating current ReRAM (accessed 03 October 2022); https://www.fujitsu.com/jp/group/fsm/en/products/reram/.

[15] Website of Anandtech. Analyzing Intel-Micron 3D XPoint: The Next Generation Non-Volatile Memory (accessed 23 July 2022); https://www.anandtech.com/show/9470/intel-and-micron-announce-3d-xpoint-nonvolatile-memory-technology-1000x-higher-performance-endurance-than-nand.

[16] Cha, E. et al. Comprehensive scaling study of NbO_2 insulator-metal-transition selector for cross point array application. *Appl. Phys. Lett.* **108**, 153502 (2016).

[17] Chen, P. S. et al. Improved bipolar resistive switching of HfO_x/TiN stack with a reactive metal layer and post metal annealing. *Japanese Journal of Applied Physics* **49**, 04DD18 (2010).

[18] Koveshnikov, S. et al. Real-time study of switching kinetics in integrated 1T/ HfOx 1R RRAM: Intrinsic tunability of set/reset voltage and trade-off with switching time. *International Electron Devices Meeting.* 20.4.1-20.4.3 (IEEE 2012).

[19] Lv, H. et al. Improvement of endurance and switching stability of forming-free CuxO RRAM. *Joint Non-Volatile Semiconductor Memory Workshop and International Conference on Memory Technology and Design.* 52-53 (IEEE 2008).

[20] Mehonic, A. et al. Silicon Oxide (SiOx): A Promising Material for Resistance Switching? *Adv. Mater.* **30**, 1801187 (2018).

[21] Chen, A. A review of emerging non-volatile memory (NVM) technologies and applications. *Solid-State Electronics* **125**, 25–38 (2016).

[22] Lanza, M. et al. Standards for the characterization of endurance in resistive switching devices. *ACS nano* **15**, 17214–17231 (2021).

[23] Lee, H. Y. et al. Low power and high speed bipolar switching with a thin reactive Ti buffer layer in robust HfO2 based RRAM. *IEDM*, 1–4 (2018).

[24] Vianello, E. et al. Sb-doped GeS2 as performance and reliability booster in Conductive Bridge RAM. *IEDM*, 31.5.1–31.5.4 (2012).

[25] Kim, K. et al. Nanoscale resistive memory with intrinsic diode characteristics and long endurance. *Appl. Phys. Lett.* **96**, 053106 (2010).

[26] Wei, Z. et al. Highly reliable TaOx ReRAM and direct evidence of redox reaction mechanism. *IEDM*, 1–4 (2008).

[27] Yang, J. J. et al. High switching endurance in TaO_x memristive devices. *Appl. Phys. Lett.* **97**, 232102 (2010).

[28] Wiefels, S. et al. Impact of the ohmic electrode on the endurance of oxide-based resistive switching memory. *IEEE transactions on electron devices* **68**, 1024-1030 (2021).

[29] Kempen, T. et al. 50x endurance improvement in TaOx RRAM by extrinsic doping. Proc. IEEE International Memory Workshop (IMW), 16−19 (2021).

[30] Merced-Grafals, E. J. et al. Repeatable, accurate, and high speed multi-level programming of memristor 1T1R arrays for power efficient analog computing applications. *Nanotechnology* **27**, 365202 (2016).

[31] You, T. et al. Exploiting memristive $BiFeO_3$ bilayer structures for compact sequential logics. *Adv. Funct. Mater.* **24**, 3357−3365 (2014).

[32] Yan, Z., Guo, Y., Zhang, G. & Liu, J. M. High-performance programmable memory devices based on co-doped $BaTiO_3$. *Adv. Mater.* **23**, 1351−1355 (2011).

[33] Oligschlaeger, R., Waser, R., Meyer, R., Karthäuser, S. & Dittmann, R. Resistive switching and data reliability of epitaxial (Ba, Sr)TiO_3 thin films. *Appl. Phys. Lett.* **88**, 042901 (2006).

[34] Son, J. et al. Electrical stabilities and memory mechanisms of organic bistable devices fabricated utilizing a poly (3, 4-ethylene-dioxythiophene): poly (styrenesulfonate) layer with a poly (methyl methacrylate) buffer layer. *Appl. Phys. Lett.* **100**, 99 (2012).

[35] Kim, Y. et al. Bi-layered RRAM with unlimited endurance and extremely uniform switching. *VLSI*, 14−16 (2011).

[36] Jiang, H. et al. Sub-10 nm Ta channel responsible for superior performance of a HfO_2 memristor. *Scientific reports* **6**, 1−8 (2016).

[37] Chand, U. et al. Suppression of endurance degradation by utilizing oxygen plasma treatment in HfO_2 resistive switching memory. *Applied Physics Letters* **106**, 153502 (2015).

[38] Zhang, B. et al. Conjugated Polymer-Grafted reduced graphene oxide for nonvolatile rewritable memory. *Chemistry–A European Journal* **17**, 13646−13652 (2011).

[39] Kim, H.-D., An, H.-M., Lee, E. B. & Kim, T. G. Stable bipolar resistive switching characteristics and resistive switching mechanisms observed in aluminum nitride-based ReRAM devices. *IEEE transactions on electron devices* **58**, 3566−3573 (2011).

[40] Shang, J. et al. Thermally stable transparent resistive random access memory based on all-oxide heterostructures. *Adv. Funct. Mater.* **24**, 2171−2179 (2014).

[41] Choi, B. J. et al. Electrical performance and scalability of Pt dispersed SiO_2 nanometallic resistance switch. *Nano letters* **13**, 3213−3217 (2013).

[42] Wang, M. et al. Robust memristors based on layered two-dimensional materials. *Nature Electronics* **1**, 130-136 (2018).

[43] Kim, H.-D., An, H.-M., Seo, Y. & Kim, T. G. Transparent resistive switching memory using ITO/AlN/ITO capacitors. *IEEE Electron Device Lett.* **32**, 1125−1127 (2011).

[44] Huang, C.-Y., Huang, C.-Y., Tsai, T.-L., Lin, C.-A. & Tseng, T.-Y. Switching mechanism of double forming process phenomenon in ZrO_x/HfO_y bilayer resistive switching memory structure with large endurance. *Appl. Phys. Lett.* **104**, 062901 (2014).
[45] Chang, K.-C. *et al.* Characteristics and mechanisms of silicon-oxide-based resistance random access memory. *IEEE Electron Device Lett.* **34**, 399−401 (2013).
[46] Zhao, X. *et al.* Confining cation injection to enhance CBRAM performance by nanopore graphene layer. *Small* **13**, 1603948 (2017).
[47] Lai, Y.-C. *et al.* Transferable and Flexible Label-Like Macromolecular Memory on Arbitrary Substrates with High Performance and a Facile Methodology. *Advanced Materials* **25**, 2733−2739, doi:https://doi.org/10.1002/adma.201205280 (2013).
[48] Yoshida, C., Tsunoda, K., Noshiro, H. & Sugiyama, Y. High speed resistive switching in Pt⁄ TiO_2⁄ TiN film for nonvolatile memory application. *Appl. Phys. Lett.* **91**, 223510 (2007).
[49] Seong, D.-j., Jo, M., Lee, D. & Hwang, H. HPHA Effect on Reversible Resistive Switching of Pt⁄ Nb-Doped $SrTiO_3$ Schottky Junction for Nonvolatile Memory Application. *Electrochemical and solid-state letters* **10**, H168 (2007).
[50] Yoong, H. Y. *et al.* Epitaxial ferroelectric $Hf_{0.5}Zr_{0.5}O_2$ thin films and their implementations in memristors for brain-inspired computing. *Advanced Functional Materials* **28**, 1806037 (2018).
[51] Kund, M. et al. Conductive-Bridging RAM (CBRAM): An Emerging Non-Volatile Memory Technology Scalable to Sub 20 nm. *IEDM*, 754−757 (2005).
[52] Tsurumaki, A., Yamada, H. & Sawa, A. Impact of Bi deficiencies on ferroelectric resistive switching characteristics observed at p-type Schottky-like $Pt/Bi_{1-\delta}FeO_3$ interfaces. *Adv. Funct. Mater.* **22**, 1040-1047 (2012).
[53] Chiu, F.-C., Li, P.-W. & Chang, W.-Y. Reliability characteristics and conduction mechanisms in resistive switching memory devices using ZnO thin films. *Nanoscale research letters* **7**, 1−9 (2012).
[54] Tsai, T.-M. *et al.* Bipolar resistive RAM characteristics induced by nickel incorporated into silicon oxide dielectrics for IC applications. *IEEE electron device letters* **33**, 1696-1698 (2012).
[55] Midya, R. *et al.* Anatomy of Ag/Hafnia-based selectors with 1010 nonlinearity. *Adv. Mater.* **29**, 1604457 (2017).
[56] Hsu, C. et al. Homogeneous barrier modulation of TaOx/TiO2 bilayers for ultra-high endurance three-dimensional storage-class memory. *Nanotechnology* **25**, 165202 (2014).
[57] Lee, M. et al. A fast, high-endurance and scalable non-volatile memory device made from asymmetric Ta2O5−x/TaO2−x bilayer structures. *Nat. Mat.* **10**, 625–630 (2011).
[58] Jo, H. et al. Cross-point resistive RAM based on field-assisted superlinear threshold selector. *IEEE Electron Device Lett*. **34**, 399−401 (2013).
[59] Kozicki, M. et al. Nanoscale Memory Elements Based on Solid-State Electrolytes. *IEEE Trans. Nanotechnol*. **4**, 331−338 (2005).
[60] Chen, Y. et al. Endurance/retention trade-off on HfO2/Metal cap 1T1R bipolar RRAM. *IEEE Trans. Electron Devices* **60**, 1114−1121 (2013).
[61] Chen, Y. et al. Balancing SET/RESET Pulse for > 1010 Endurance in HfO2/Hf 1T1R Bipolar RRAM. *IEEE Trans. Electron Devices* **59**, 3243−3249 (2012).
[62] Lv, H. et al. Evolution of conductive filament and its impact on reliability issues in oxide-electrolyte based resistive random access memory. *Sci. Rep.* **5**, 7764 (2015).
[63] Lee, M. et al. A plasma-treated chalcogenide switch device for stackable scalable 3D nanoscale memory. *IEDM*, 33−35 (2012).
[64] Cao, R. et al. Improvement of Device Reliability by Introducing a BEOL-Compatible TiN Barrier Layer in CBRAM. *IEEE Electron Device Lett.* **38**, 1371−1374 (2017).
[65] Chien, W. et al. Unipolar switching behaviors of RTOWOX RRAM. *IEEE Electron Device Lett.* **31**, 126−128 (2010).
[66] Aratani, K. et al. A Novel Resistance Memory with High Scalability and Nanosecond Switching. *IEDM*, 783−786 (2017).
[67] Chen, Y. et al. Hydrogen induced redox mechanism in amorphous carbon resistive random access memory. *Nanoscale Res. Lett.* **9**, 52 (2014).

[68] Govoreanu, B. et al. 2011 10×10 nm2 Hf/HfOx crossbar resistive RAM with excellent performance, reliability and low-energy operation. *IEDM*, 729−732 (2011).
[69] Chen, C. et al. Stack optimization of oxide-based RRAM for fast write speed (<1 ls) at low operating current (<10 lA). *Solid-State Electron*. **125**, 198−203 (2016).
[70] Lee, H. et al. Low-power and nanosecond switching in robust hafnium oxide resistive memory with a thin Ti cap. *IEEE Electron Device Lett.* **31**,44−46 (2010).
[71] Chien, W. et al. A forming-free WOX resistive memory using a novel self-aligned field enhancement feature with excellent reliability and scalability. *IEDM*, 440−443 (2010).
[72] Baek, I. et al. Highly Scalable non-volatile resistive memory using simple binary oxide driven by asymmetric unipolar voltage pulses. *IEDM* 587−590 (2004).
[73] Lee, J. et al. Diode-less Nano-scale ZrOx/HfOx RRAM Device with Excellent Switching Uniformity and Reliability for High-density Cross-point Memory Applications. *IEDM* 452−455 (2010).
[74] Seong, D. et al. Effect of oxygen migration and interface engineering on resistance switching behavior of reactive metal/polycrystalline Pr0.7Ca0.3MnO3 device for nonvolatile memory applications. *IEDM* 101−104 (2009).
[75] Belmonte, A. et al. 90nm W\Al2O3\TiW\Cu 1T1R CBRAM cell showing low-power, fast and disturb-free operation. *Proc. IEEE International Memory Workshop (IMW)*, 26−30 (2013).
[76] Emerging non-volatile memory 2021 (market analysis report), Yole Development (accessed 03 October 2022); https://s3.i-micronews.com/uploads/2021/02/YINTR21218-emerging-Non-Volatil-Memory-2021-sample.pdf
[77] Gallo, M. L. et al. Mixed-precision in-memory computing. *Nat. Electron*. **1**, 246–253 (2018).
[78] Woods, W. & Teuscher. C. Approximate vector matrix multiplication implementations for neuromorphic applications using memristive crossbars. Proc. IEEE/ACM Int. Symp. Nanoscale Archit. (NANOARCH) 103–108 (2017).
[79] Hu, M. et al. Dot-product engine for neuromorphic computing: programming 1T1M crossbar to accelerate matrix-vector multiplication. *ACM/EDAC/IEEE Design Automation Conference (DAC)*, 1–6 (2016),
[80] Serb, A. et al. Practical determination of individual element resistive states in selectorless RRAM arrays. *IEEE Transactions on Circuits and Systems* **63**, 827–835. (2016).
[81] Cai, F., Correll, J. M., Lee, S. H., Lim, Y., Bothra, V., Zhang, Z., Flynn, M. P., & Lu, W. D. (2019). A fully integrated reprogrammable memristor–CMOS system for efficient multiply–accumulate operations. Nature Electronics, 2(7), 290–299. https://doi.org/10.1038/s41928-019-0270-x
[82] Roldan, J. B. et al. Spiking neural networks based on two-dimensional materials. *npj 2D Mater. Appl.* **6**, 63 (2022).
[83] Yang, J. et al. Memristive devices for computing. *Nat. Nanotech.* **8**, 13–24 (2013)
[84] Merolla, P. A. et al. A million spiking-neuron integrated circuit with a scalable communication network and interface. *Science* **345**, 668–673 (2014)
[85] Diehl, P. U. et. Unsupervised learning of digit recognition using spike-timing-dependent plasticity. *Front. Comput. Neurosci.* **9**, 1662–5188 (2015).
[86] LeCun, Y., Cortes, C. & Burges, C. J. C. The MNIST database of handwritten digits. (accessed 03 October 2022); http://yann.lecun.com/exdb/mnist
[87] Stimberg, M et al. Brian2, an intuitive and efficient neural simulator. *Elife* **8**, e47314 (2019).
[88] K. Gerstner, W.M. Kistler, "Spiking neuron models: Single neurons, Populations, Plasticity", Cambridge University Press, 2002.
[89] Zhang, W. et al., The other side of the engram: experience-driven changes in neuronal intrinsic excitability. *Nat. Rev. Neuroscience* **411**, 4, 885–900 (2003)
[90] Querlioz, D. et al., Immunity to device variations in a spiking neural network with memristive nanodevices. *IEEE Trans. Nanotechnology* **12**, 288–295, (2013)
[91] Gerstner, W. et al., A neuronal learning rule for sub-millisecond temporal coding. *Nature* **386**, 76-78, (1996)
[92] Kempter, R. et al., Hebbian learning and spiking neurons. *Physical Review E* **59**, 4498–4514, (1999).

[93] Morrrison, A. et al. Spike-timing-dependent plasticity in balanced random networks. *Neural Computation* **19**, 1437–1467 (2007).
[94] Chicca, E. et al. Neuromorphic electronic circuits for building autonomous cognitive systems. *Proc. IEEE*, **102**, 1367–1388 (2014).
[95] Miranda, E. Compact model for the major and minor hysteretic I–V Loops in nonlinear memristive devices. *IEEE Trans. Nanotechnol*. **14**, 787–789 (2015).
[96] Aguirre, F. L. et al. Application of the quasi-static memdiode model in cross-point arrays for large dataset pattern recognition. *IEEE Access* **8**, 202174–202193 (2020).
[97] Zahari, F. et al. Pattern recognition with TiOx-based memristive devices. *AIMS Mater. Sci*. **2**, 3, (2015).
[98] Hansen, M. et al. Double-barrier memristive devices for unsupervised learning and pattern recognition. *Front. Neurosci*. **11**, 91 (2017).
[99] Valentian, A. et al. Fully integrated spiking neural network with analog neurons and RRAM synapses. *IEDM* 14.3.1-14.3.4 (2019).
[100] Ishii, M. et al. On-chip trainable 1.4M 6T2R PCM synaptic array with 1.6K stochastic LIF neurons for spiking RBM. *IEDM* 14.2.1-14.2.4 (2019).
[101] Diehl, P. U. et al. Conversion of artificial recurrent neural networks to spiking neural networks for low-power neuromorphic hardware. Proc. Int. Jt. Conf. Neural Networks, 1−8 (2015).
[102] O'Brien K. P. et al. Advancing 2D monolayer CMOS through contact, channel and interface engineering. *IEEE International Electron Devices Meeting (IEDM)* 7.1.1-7.1.4 (IEEE 2021).
[103] Lin, Y. et al. Contact engineering for high-performance N-type 2D semiconductor transistors, *IEEE International Electron Devices Meeting (IEDM)* 37.2.1-37.2.4 (IEEE 2021).
[104] C. Dorow et al. Advancing monolayer 2D NMOS and PMOS transistor integration from growth to van der Waals interface engineering for ultimate CMOS scaling. *Symposium on VLSI Technology*. 1-2 (2021).
[105] Chou, A. S. et al. Antimony semimetal contact with enhanced thermal stability for high performance 2D electronics. *IEEE International Electron Devices Meeting (IEDM)* 7.2.1–7.2.4 (IEEE, 2021).
[106] Lin, D. et al. Dual gate synthetic WS2 MOSFETs with 120 μS/μm Gm 2.7 μF/cm2 capacitance and ambipolar channel. *IEEE International Electron Devices Meeting (IEDM)* 3.6.1–3.6.4 (IEEE 2020).
[107] Wu, X. et al. Dual gate synthetic MoS2 MOSFETs with 4.56μF/cm2 channel capacitance, 320μS/μm Gm and 420 μA/ μm Id at 1V Vd/100nm Lg. *IEEE International Electron Devices Meeting (IEDM)* 7.4.1-7.4.4 (IEEE 2021).
